# Low‐Power Perovskite‐Based Memristors Enable Fused Reservoir Computing and Neuromorphic Vision with Highly Accurate Color Perception

**DOI:** 10.1002/smll.202508167

**Published:** 2025-11-28

**Authors:** Panagiotis Bousoulas, Spyros Orfanoudakis, Leonidas Tsetseris, Chalalampos Tsioustas, Stefania Skorda, Alexandros El Sachat, Polychronis Tsipas, Athanassios G. Kontos, Thomas Stergiopoulos, Dimitris Tsoukalas

**Affiliations:** ^1^ Department of Physics School of Applied Mathematical and Physical Sciences National Technical University of Athens Iroon Polytechniou 9 Zographou Athens 15780 Greece; ^2^ Institute of Nanoscience and Nanotechnology NCSR Demokritos Athens 15341 Greece

**Keywords:** artificial synapses, diffusion, heterostructures, memristor, neuromorphic computing, optoelectronic perovskites, reservoir computing

## Abstract

Integrating multicolor perception with neuromorphic vision systems, capable of emulating the procedures of image detection, storage, and local processing, represents a significant advancement in artificial visual technologies. However, challenges related to data fusion, system complexity, and stability must be addressed to fully realize the potential of this technology. In this work, a low‐dimensional/three‐dimensional (LD/3D) halide perovskite heterostructure consisting of Ag/LD perovskitoid/3D CsFAMA/ITO is fabricated, demonstrating excellent stability for 2 months combined with the co‐existence of two switching modes, namely volatile and non‐volatile. The former mode is leveraged to construct the nodes of the reservoir computing architecture, where the fusion rate of the electrical and optical signals is examined to achieve maximum recognition accuracy of multicolor handwritten MNIST images (84%). An ultra‐low power consumption of 400 fJ per synaptic weight change is also recorded during red light irradiation. By combining experiments with different top electrode materials and extensive Density Functional Theory calculations on metal atom diffusion and clustering in the materials of interest, key atomic scale processes are identified that underlie the switching behavior and lead to improved memory performance. The ability of the proposed device configuration to accurately carry out multimodal recognition tasks opens new possibilities for realizing biomimetic systems.

## Graphical abstract

The development of low‐power multicolor neuromorphic computing systems could endow artificial vision machines with optical perception and processing capabilities in a way similar to biological systems, such as the human eye. Along these lines, perovskite‐based heterostructures emerge as a promising solution since their mixed ionic/electronic properties can be leveraged to build powerful in‐memory computing systems.

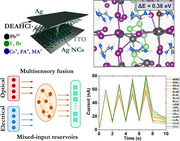

## 1 Introduction

The development of real‐world artificial intelligence (AI) functionalities calls for the design and operation of novel electronic devices located at the edge, which will enable the processing of various types of input sensory data (i.e. visual, auditory) in an energy‐efficient manner.^[^ ^1^ ^]^ The already existing AI hardware configurations cannot address this goal due to the “memory wall” problem: the collected data have to be constantly transferred between distinct memory and compute units, a significant caveat which inevitably leads to high power consumption and high latency.^[^ ^2^ ^]^ Moreover, conventional systems produce a huge amount of redundant data when they deal with challenging cognitive processes. In most cases, the raw data are sent to a computational cloud for further signal processing and then, the final decisions are transferred back to the AI system.^[^ ^3^ ^]^ This enormous computational effort to collect and return the information packages between the AI system and the cloud requires a constant data bandwidth and inevitably limits the performance. The associated latency problem has also a direct impact on the development of ultrafast responsive systems.

To effectively address these technological issues, novel computing architectures have been devised. Among the various computing concepts, neuromorphic computing arises as an ideal candidate due to its superb capabilities for in‐memory computing, which permits low latency and power consumption, as well as fault‐tolerant processing capabilities.^[^ ^4^ ^]^ The utilization of an in‐memory computing configuration offers a unique pathway in attaining brain‐level information processing efficiency since the costly data movement that takes place within the conventional von Neumann architecture is circumvented. Resistive random‐access memory (RRAM) is regarded as an emerging memory technology that, compared to the standard on‐chip static random access memory (SRAM), has the advantages of enhanced integration density, low leakage current dissipation, and improved analog programmability properties.^[^ ^5^ ^]^ For this reason, RRAM structures have been extensively investigated for the implementation of large‐scale and low‐power compute‐in‐memory systems. Moreover, the analog computing capabilities of memristive arrays provide ideal opportunities to interface them with modern sensing elements. In particular, the physical ability of memristors to operate with analog signals alleviates the need to integrate analog‐to‐digital and digital‐to‐analog (ADC/DAC) converters, which are energy‐hungry and normally consume most of the energy in a mixed‐signal neural network.^[^ ^6^ ^]^ The memristive devices could also be directly leveraged for the low‐level decision‐making processes by exploiting the various switching modes (i.e. threshold and bipolar).^[^ ^7^, ^8^, ^9^ ^]^

Vision is arguably the most critical of the five senses for humans due to its profound impact on how we interact with and understand the world. However, challenges arising from the complexity of visual data such as variations in lighting, perspective, and occlusions, combined with the enormous computational demands, have hindered the further development of artificial vision systems. Neuromorphic vision represents a transformative approach to visual processing, inspired by the unique architecture and function of the brain. By emulating the way biological systems perceive and process visual information, neuromorphic vision systems can offer comparative advantages in speed, power efficiency, and adaptability.^[^ ^10^, ^11^, ^12^ ^]^ Various multimodal sensory systems have been also reported in the literature using discrete sensory and processing elements to emulate the behavior of their biological counterparts.^[^ ^13^, ^14^, ^15^, ^16^, ^17^ ^]^ Although the integration of sensing and processing properties has been recently achieved by single devices, it has relied on complex material configurations and power consumption remains relatively high.^[^ ^18^, ^19^, ^20^, ^21^, ^22^, ^23^, ^24^ ^]^ In addition, the influence of the different fused input signals on the sensing accuracy has not been systematically examined.

To meet the above‐mentioned stringent demands, multifunctional materials with intriguing properties are urgently required. To this end, hybrid organic‐inorganic, metal‐halide perovskites are a versatile and highly promising class of materials with unique properties. High‐quality crystalline perovskite films can be fabricated using simple, low‐cost methods, such as solution processing, spin‐coating, or printing techniques, which renders them attractive for large‐scale production.^[^ ^25^, ^26^ ^]^ These films present only few defects that are relatively benign, suggesting that their electronic properties are not significantly degraded by imperfections in the crystal lattice.^[^ ^27^ ^]^ Additionally, perovskites exhibit mixed electronic/ionic conduction properties.^[^ ^28^, ^29^ ^]^ This property can affect materials performance in devices, leading to the manifestation of hysteresis phenomena with low power consumption. Various efforts have been also devoted to understanding the physical origins of the hysteresis effect including the formation of conducting filaments (CFs) composed of metal cations in the majority of cases.^[^ ^30^ ^]^ In parallel, several other mechanisms have been suggested, such as the participation of iodide and bromide ions in the switching process,^[^ ^31^, ^32^ ^]^ as well as the manifestation of a charge trapping/detrapping mechanism,^[^ ^33^ ^]^ and the modulation of the Schottky barrier width.^[^ ^34^ ^]^ The impact of the top electrode (TE) in typical 3D methylammonium lead iodide (MAPbI_3_) memristors has been also examined, providing valuable insights into the metal's electrochemical activity.^[^ ^35^ ^]^ Nonetheless, to acquire reconfigurable sensing and neuromorphic functionalities in an energy‐efficient manner and boost the implementation of halide perovskites in practical neuromorphic applications, the reproducibility of the switching effect and the stability of the perovskite material itself against environmental factors should be enhanced. To address this, buffer layers^[^ ^36^, ^37^, ^38^ ^]^ and, more importantly, two‐dimensional (2D)/3D perovskite heterostructures^[^ ^39^, ^40^, ^41^, ^42^ ^]^ have been adopted; in the latter case, a 2D layer is formed on top of the 3D perovskite, protecting the sensitive surface and fine‐tuning the electronic properties of the heterostructure.^[^ ^43^ ^]^

Along these lines, in this work, a conventionally performing perovskite, that is the PbI_2_‐excessive cesium‐formamidinium‐methylammonium (Cs_0.05_FA_0.9_MA_0.05_PbI_2.95_Br_0.05_) halide perovskite with a 3D structure was adopted. The perovskite was combined with 2‐diethylaminoethanethiol hydrochloride (DEAHCl) ontop, resulting in the formation of a low dimensional (LD) perovskitoid structure. This modification leads to robust memristors with 100% device yield, in terms of working devices, and stable operation for more than 2 months, whereas the majority of the reported devices fail within a short period. The role of the DEAHCl additives, as well as the diffusion and clustering of Ag species (which enable the CF formation) were thoroughly investigated through Density Functional Theory (DFT) calculations. Conductive Atomic Force Microscopy (C‐AFM) measurements were also performed to investigate the possible formation of percolating conducting filaments (CFs). The devices operated under the application of low switching voltages of 100 mV, yielding a power consumption of ≈5 pJ per synaptic weight change. An even smaller power consumption of 400 fJ was recorded under red light illumination, comparable to that of biological neurons and the lowest reported for CsFAMA‐based memristors. Various TE materials with low (ITO, TiN), intermediate (W), and high (Ag) chemical reactivity, as well as inert electrodes (Au) were used in order to elaborate on the dominant switching mechanism. Both volatile and non‐volatile responses were recorded, which were leveraged to emulate various synaptic functionalities under the application of both electrical and optical stimuli. The ability to tune the relaxation time of our prototypes by using top‐gate biasing or applying light pulses through the transparent bottom electrode (BE), called heterosynaptic plasticity,^[^ ^44^ ^]^ enabled the implementation of the reservoir computing (RC) architecture with fused modes. The effect of the different fused modes on the recognition accuracy of colored handwritten numbers was systematically analyzed. In particular, a recognition accuracy of 84% was demonstrated using the accurate color perception properties of the proposed devices in recognizing multicolor handwritten MNIST images. Finally, outstanding multimodal properties were demonstrated through simulations of simultaneous visual and audio signal recognition, highlighting the ability to perform biological‐like multisensory operations with an accuracy exceeding 82%. The development of such a system could significantly enhance artificial vision capabilities, enabling artificial neuromorphic systems to perceive and process visual information in a way similar to biological systems, such as the human eye. The ability to process multicolor inputs directly could improve color recognition, object detection, and even depth perception. A multicolor cognitive RC system that directly processes optical inputs without additional hardware components represents a significant step forward in creating more integrated and powerful artificial vision systems. By eliminating the need for separate sensors and processors, it not only overcomes traditional hardware limitations but also enables the development of more compact and efficient artificial vision systems.

## 2 Results and Discussion

### 2.1 Material Properties

**Figure 1:**
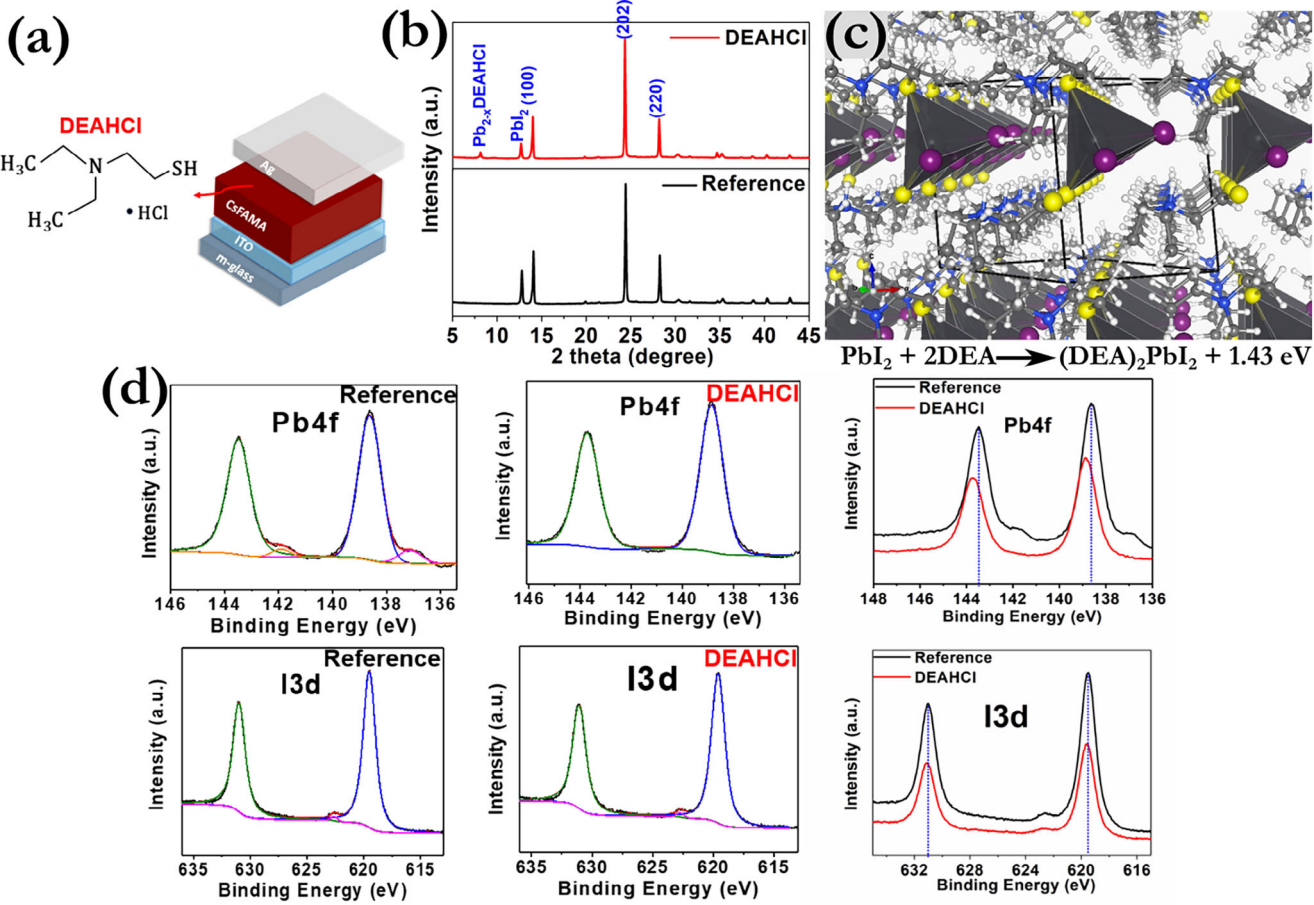
a) Schematic illustration of the device configuration. b) XRD diffractograms of the ITO/CsFAMA and ITO/CsFAMA/DEAHCl films. c) DFT results for a low‐dimensional (1D) crystal structure formed through the depicted exothermic reaction of DEA molecules with a PbI_2_ crystal (Pb: large gray, C: gray, H: white, N: blue, S: yellow, I: purple spheres). The black lines depict the unit cell. In this structure, the DEA makes a bond through its S end to a Pb atom (the corresponding H atom is transferred to the N site of the DEA). d) Pb4f and I3d XPS spectra of the CsFAMA and CsFAMA/DEAHCl films with the corresponding peaks after fitting analysis.

To evaluate the impact of DEAHCl on the structural properties of the perovskite structure, several material characterization techniques were used. Our initial step was to validate the structure of the employed perovskite and examine the changes that occur after post‐treatment (**Figure** **1**a), utilizing X‐ray diffraction (XRD) measurements (Figure 1b). All the peaks, corresponding to the *α*‐phase of CsFAMA, were identical for both reference and post‐treated films.^[^ ^45^ ^]^ The clear XRD peak of PbI_2_ (significantly reduced compared to the reference film) suggests the existence of layered PbI_2_, the role of which is to passivate the grain boundaries and surfaces. Furthermore, a new peak at 8.1° was observed in the post‐treated films, which has been previously assigned to the formation of a low‐dimensional perovskitoid ontop of the conventional 3D CsFAMA perovskite.^[^ ^46^, ^47^, ^48^ ^]^ This perovskitoid could form due to the chemical interaction of DEAHCl with the excess PbI_2_ (4%), which we intentionally incorporate in the precursor solution to enhance crystallization.^[^ ^49^ ^]^ The presence of the perovskitoid was further confirmed when depositing DEAHCl ontop of a pure PbI_2_ film; a very prominent peak at similarly low degrees in the XRD was observed (Figure S1, Supporting Information).

From the high‐resolution X‐ray photoelectron spectroscopy (XPS) spectra of the Pb 4f core levels in both the reference and the passivated perovskite films, distinct and sharp peaks, located at specific binding energies at 143.4 and 138.7 eV, were detected, identified as 4f5/2 and 4f7/2, respectively, in CsFAMA perovskite (Figure 1d). Post‐treatment with DEAHCl induced significant shifts of ≈+0.3 eV to lower binding energies suggesting interaction between uncoordinated Pb^2+^ and DEAHCl.^[^ ^50^, ^51^ ^]^ On the other hand, only minor shifts of ≈+0.1 eV were observed for the I3d spectra (Figure 1d), suggesting that DEAHCl mainly interacts with Pb, as will be further demonstrated by the DFT calculations in the next section. This analysis focuses on establishing the role of DEAHCl in modifying the interfacial chemistry, particularly through its interaction with undercoordinated Pb species at the perovskite surface.

**Figure 2:**
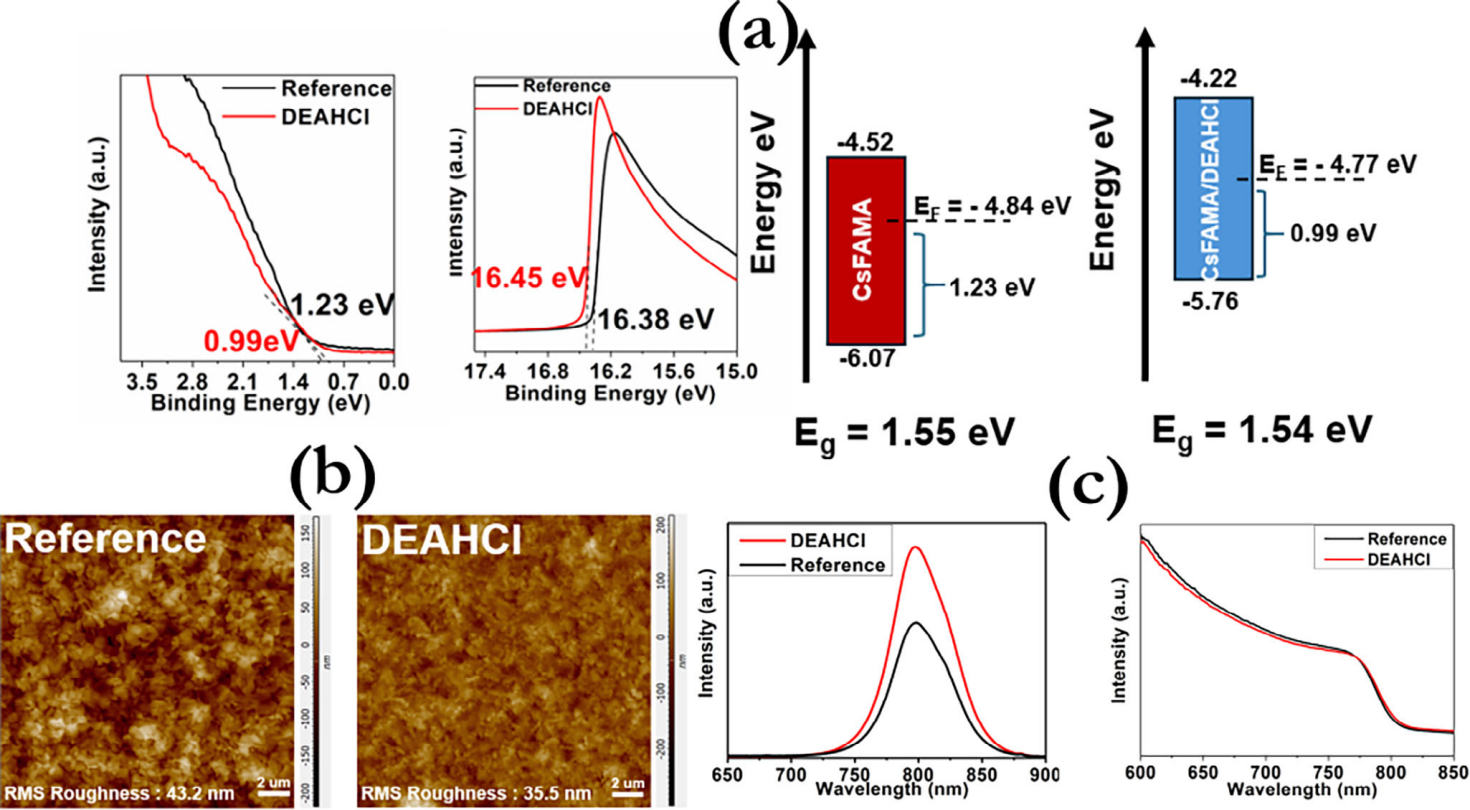
a) UPS spectra and energy alignment of the ITO/CsFAMA and ITO/CsFAMA/DEAHCl films. b) Contact Mode AFM images of the CsFAMA and CsFAMA/DEAHCl perovskite films c) Steady‐state PL spectra and UV–vis spectra of bare CsFAMA with and without DEAHCl passivation.

The utilization of ultraviolet photoelectron spectroscopy (UPS) (**Figure** **2**a) provides more conclusive pieces of evidence regarding the impact of DEAHCl on the electron energy diagram of the CsFAMA. The valence band edge (V_B_) and the work function (W_F_) of the perovskite films were determined using the equations W_F_ = hν‐E_cutoff_ and E_VB_ = E_F_ ‐ E_onset_, respectively. The cutoff and onset energies of the secondary electrons for the different perovskite films were obtained directly from the UPS spectra.^[^ ^52^, ^53^ ^]^ Estimated values of W_F_, equal to 4.84 and (4.77) eV, E_VB_, equal to ‐1.23 and (‐0.99) eV, as well as ionization energies, I_E_ = W_F_ + E_VB_, equal to 6.07 and (5.76) eV were calculated for bare and passivated films (in parenthesis), respectively. Accordingly, conduction band minima were determined as E_CB_ = (E_VB_ + Eg), equal to +0.32 and (+0.55) eV, whereas E_g_ was estimated from the Tauc plots (Figure S2, Supporting Information). The above changes in the electronic characteristics indicate that DEAHCl modification alters the band structure of the perovskite layer in conjunction with the role of passivating ligands in other studies.^[^ ^49^ ^]^

Atomic force microscopy (AFM) measurements were conducted on both reference and DEAHCl‐modified perovskite films to investigate their surface topography (Figure 2b). As can be observed, DEAHCl modification significantly reduced the surface RMS roughness from 42 nm down to 35 nm, when compared to the untreated films. Moreover, small grains on the perovskite surface can be distinguished.^[^ ^39^, ^54^ ^]^ More details regarding the structural properties and stability of the proposed perovskite structures can be found in Figure S3 (Supporting Information).

As shown in Figure 2c, ultraviolet−visible (UV−vis) absorption and photoluminescence (PL) spectroscopically measurements were carried out in order to evaluate the optical properties of the reference and modified films. An increase in the PL spectra of ≈40% after the modification with DEAHCl was observed, suggesting that the radiative recombination of the film is severely enhanced, further confirming the passivating role of DEAHCl.^[^ ^55^, ^56^ ^]^ For the UV–vis spectra, a slight increase in the absorption of the DEAHCl‐passivated films was observed, supporting the interaction of DEAHCl with the perovskite,^[^ ^57^, ^58^ ^]^ which could lead to enhanced stability of the perovskite structure under ambient conditions.^[^ ^59^, ^60^ ^]^ Τhe stability of the films was also evaluated through XRD measurements (Figure S4, Supporting Information). The films were measured on the 1^st^ and 15^th^ day after being stored under ambient conditions (relative humidity of 50%). The results fully justified our previous findings: while the intensity of the PbI_2_ diffraction peak was significantly increased in the degraded CsFAMA sample after 15 days, the passivated film exhibited no structural changes, and its diffractogram remained nearly identical between the 1st and 15th day.^[^ ^48^ ^]^ All these enhanced properties of the passivated film were leveraged to fabricate robust memory devices with enhanced operational performance, as will be demonstrated in the following sections.

### 2.2 DFT Calculations

Extensive DFT calculations were performed in order to identify key atomic‐scale processes that underlie the main experimental findings on structures and memristive behavior. First, we probed the interactions between DEAHCl and the perovskite or the PbI_2_ material. We found that the DEAHCl interacts via its S atom with a surface Pb site forming a Pb‐S bond. Based on this initial finding, we investigated further the possibility that such interactions may lead to the formation of low‐dimensional materials. Indeed, as is shown in Figure S5 (Supporting Information), the DEA molecules (without the HCl moiety) can react with the perovskite to form a 2D material with Pb‐S bonds. The reaction is exothermic with an energy gain of 1.08 eV per DEA molecule (referenced to an isolated molecule). This 2D structure has a strong XRD peak at 7.84°, a value which is very close to the experimental one of 8.1°. Likewise, DEA molecules can favorably react with the layered PbI_2_ material to form the one‐dimensional (1D) structure shown in Figure 1c with an energy gain of 1.08 eV per DEA molecule. Also in this case, Pb‐S bonds are formed and a strong XRD peak appears at 8.45° (again close to the experimental value of 8.1°). Even though we do not exclude the possibility of other LD structures, the above results are fully consistent with the experimental findings on the addition of DEAHCl, in particular with regard to the formation of Pb‐S bonds, to the decrease of the XRD signal and the position of the LD‐related XRD peak.

Another set of DFT calculations targeted the behavior of Ag impurities in the bulk of the perovskite. Figure S6 (Supporting Information) depicts stable configurations of an individual Ag atom in the perovskite, as well as the transition state (TS) encountered during hopping of the impurity from one stable site to a neighboring one. The energy of the TS gives a value of 0.41 eV for the diffusion barrier for Ag, a relatively low value which indicates that Ag diffusion is activated at room temperature. We have also calculated the formation energy (1.09 eV) of the Ag impurity, i.e. the energy required to transfer a Ag atom from the bulk of a Ag crystal inside the halide perovskite. The corresponding formation energies (referenced to bulk Ag) for a Ag atom inside a PbI_2_ crystal, inside the 2D crystal shown in Figure S5 (Supporting Information), or inside the 1D crystal shown in Figure 1c are significantly higher, 1.77, 1.92, and 1.38 eV, respectively. Therefore, it is, in principle, energetically favorable for most Ag impurities to be found in the perovskite part of the device.

In addition, we probed the agglomeration of Ag impurities in the perovskite. As shown in Figure S7 (Supporting Information), stable clusters with an increasing number of Ag atoms can be formed by nucleation around a Pb atom. The corresponding binding energies are significant (in the range of 0.16–0.59 eV), making thus possible the formation of a Ag‐based CF within the perovskite. We have also investigated the insertion of Ag atoms in the layered PbI_2_ material and in the LD structures we described above. We found that Ag diffusion is facile in PbI_2_ (the calculated barrier is ≈0.32 eV and the TS is depicted in Figure S8, Supporting Information) and that Ag clustering is also possible. Hence, any Ag impurities that may end up in PbI_2_ will diffuse very fast and thus form quickly large clusters of Ag. However, these diffusion and clustering processes ought to proceed layer by layer in PbI_2_, which is not consistent with the formation of a directed CF typically found in memristive materials. Nevertheless, the formation of the LD perovskitoid can block the diffusion of Ag impurities toward the latter layered material. Indeed, results on the diffusion barrier for a Ag atom in the LD structure of Fig. 1(c) suggest a value in excess of 1.2 eV due to the highly anisotropic structure of this material (Figures S9 and S10, Supporting Information).

### 2.3 DC and AC Device Evaluation and C‐AFM Measurements

**Figure 3:**
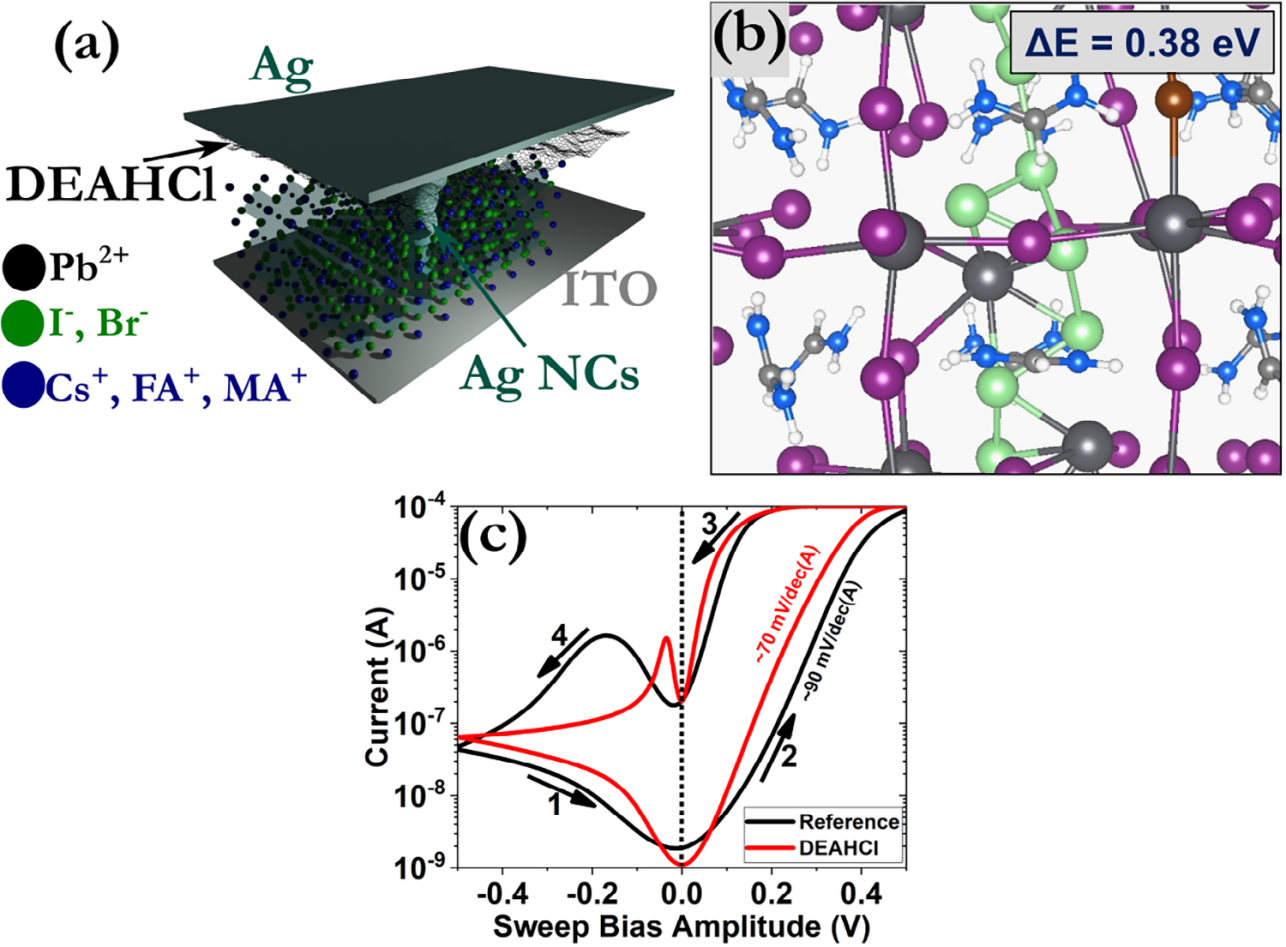
a) Structure of the proposed device configuration and the percolating CF composed of Ag nanoclusters (NCs). b) Formation of an extended chain comprising Ag atoms in a FAPbI_3‐x_Br_x_ crystal. ΔΕ is the binding energy of the last Ag atom that has been added to the cluster. c) *I*–*V* hysteresis patterns for the reference and DEAHCl samples with the application of 10 mV s^−1^. A constant I_cc_ of 100 µA was enforced for both samples. The numbers and arrows in the graph signify the switching direction. Similar hysteresis patterns were obtained by starting the voltage scans from 0 V to either positive or negative biases.

**Figure** **3**a depicts a cross‐section of the fabricated devices, whereas the extracted current–voltage (*I*–*V*) characteristics are provided in Figure 3c. No forming process was required prior to device operation and both samples exhibited bipolar switching patterns under the application of a compliance current (I_cc_) limit of 100 µA. The SET transition took place at ≈200 mV for the reference and the DEAHCl samples, but steeper switching slopes were recorded for the latter. This behavior is consistent with the DFT results that predict a relatively small diffusion barrier and formation energy for the Ag atoms in the perovskite structure when an LD layer is present ontop (Figure 3b).^[^ ^61^, ^62^, ^63^ ^]^ On the contrary, the RESET process manifests with a more abrupt pattern at ≈‐200 mV and ‐50 mV for reference and DEAHCl samples, respectively, suggesting the existence of a thermal accelerated mechanism. The smaller RESET voltage of the DEAHCl sample indicates a more controllable rupture of the CF. A switching ratio of ≈5 orders of magnitude was also recorded for both samples, which is comparable to the best performance that has been reported in the literature for mixed organic‐inorganic halide perovskites composed of CsFAMA.^[^ ^64^, ^65^, ^66^, ^67^ ^]^

**Figure 4:**
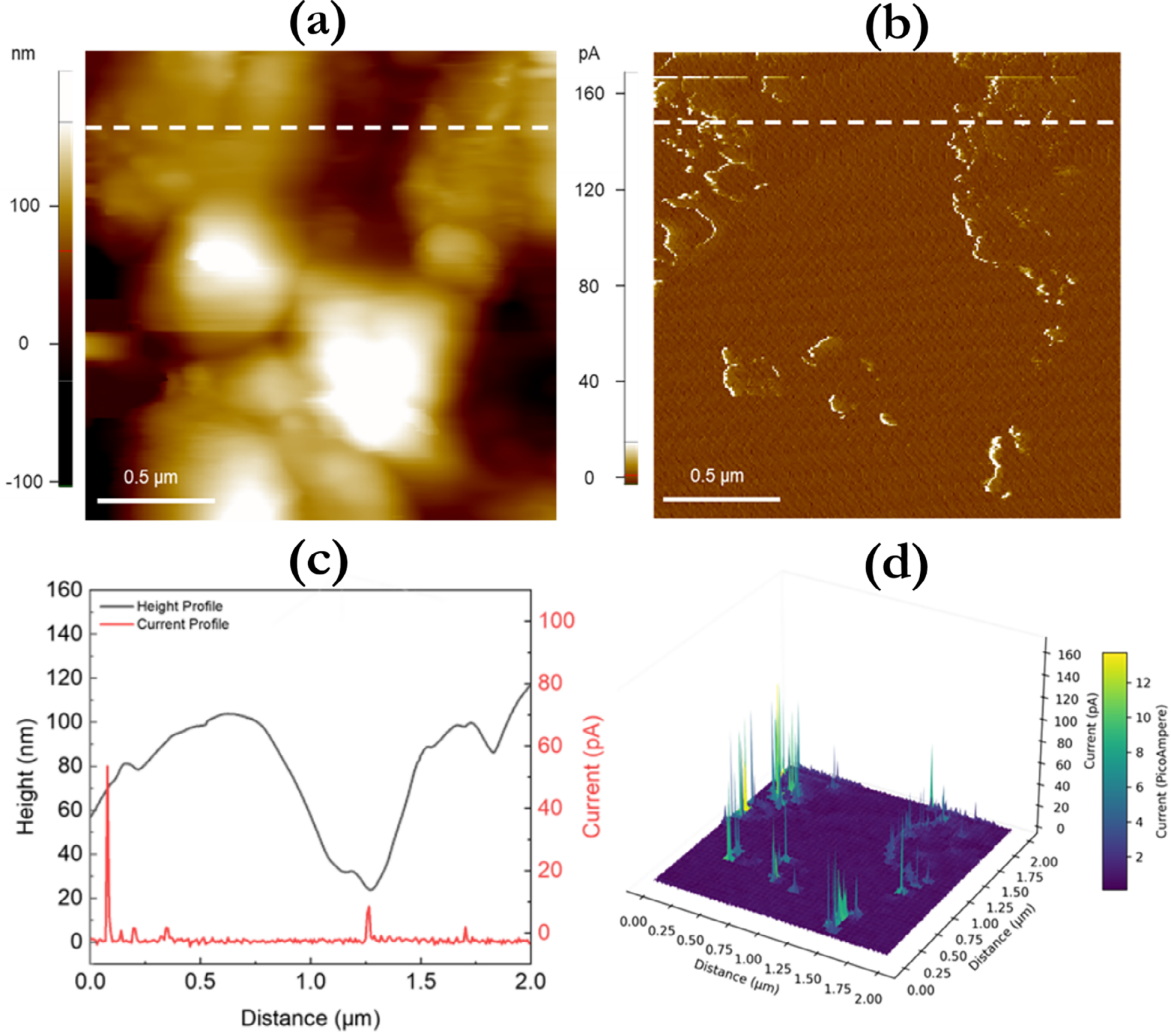
a) Structure of topography and b) current maps of the perovskite film at 1 V acquired via C‐AFM under high‐vacuum avoiding contaminants like water and hydrocarbons. c) Height and current profiles measured along the dashed white lines depicted in (a) and (b) and d) the corresponding 3D view of the current flow within the perovskite.

To investigate the potential formation of CFs within the DEAHCl samples, C‐AFM measurements were performed under high‐vacuum conditions after first programming the devices to the low resistance state (LRS) and then removing the top Ag electrode by laser ablation. The CFs were analyzed by simultaneously measuring the topography and the electric current flow at the contact point of the tip with the perovskite's top surface. **Figure** **4**a,b displays the topography and current maps of the perovskite layer by applying a constant readout bias of 1 V from the substrate with a solid Pt tip. As can be observed in Figure 4b, the CFs appear as bright (white) conductive spots, typically located around the grain boundaries of the perovskite film, while the surrounding areas remain insulating (brown background in Figure 4b). Notably, no conductive spots were detected on the Ag surface outside the laser‐ablated region, as shown in Figure S11 (Supporting Information). The height and 3D current profiles are provided in Figure 4c,d, respectively.

**Figure 5:**
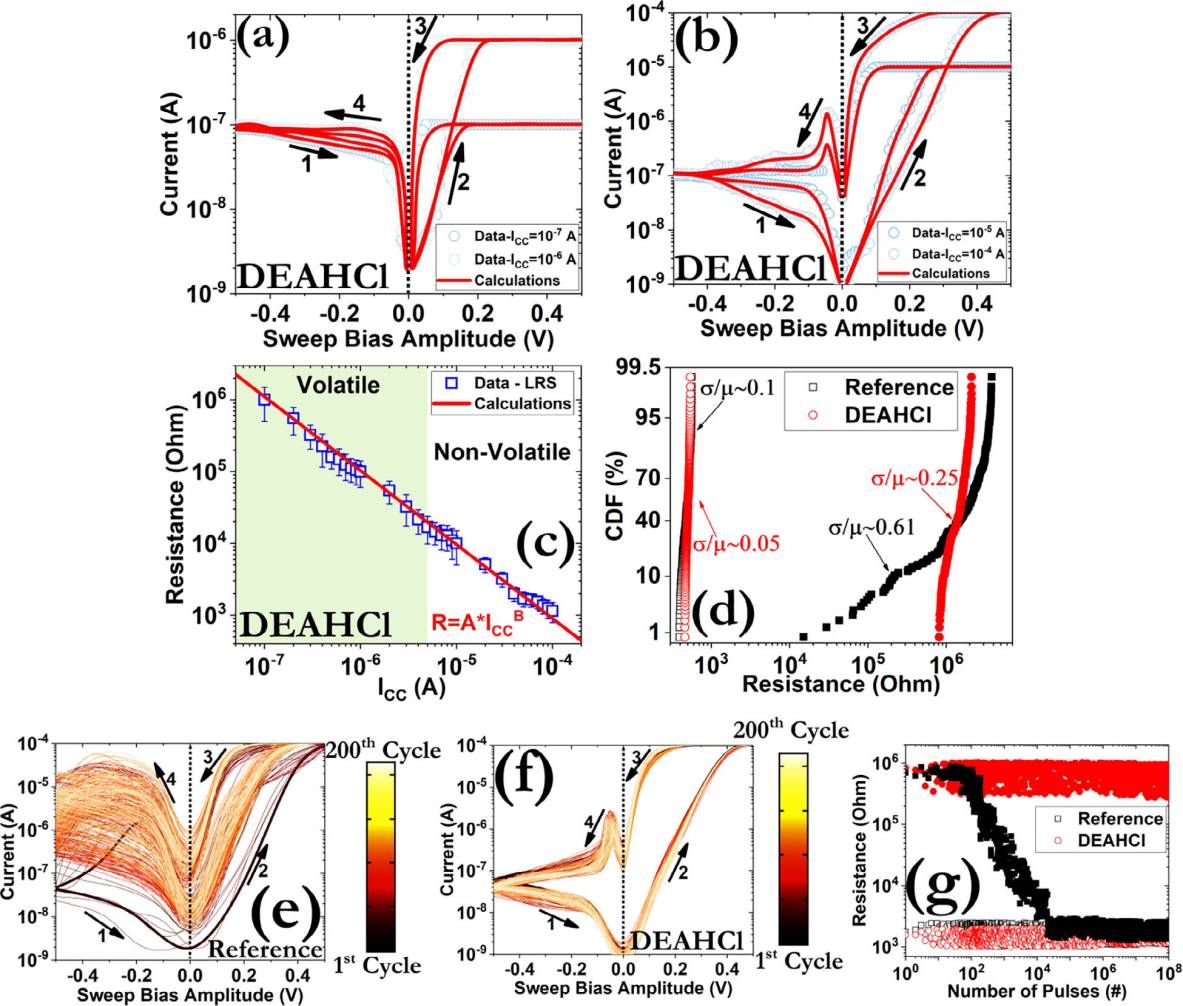
Measured and calculated current responses under the application of various I_cc_ ranging from a) 0.1–1 µA to b) 10–100 µA. c) Measured and calculated distribution of the LRS as a function of I_cc_. d) CDF plots for both samples considered in this work. The data have been collected by measuring 300 different devices on each sample. The open symbols correspond to the LRS and the filled ones to the HRS. *I*–*V* consecutive cycling behavior after the implementation of 200 direct current (DC) endurance cycles for e) reference and f) DEAHCl samples. g) Pulse endurance measurements under the application of alternative pairs of +1 V / 1 µs and −0.5 V / 1 µs square pulses. The read‐out process was carried out using square pulses of 100 mV / 1 µs.

The existence of dual switching modes, namely volatile and non‐volatile, was also observed by varying the I_cc_ (**Figure** **5**a,b), which has not been previously reported for CsFAMA‐based memristors (Table S2, Supporting Information). The dependence of LRS from the enforced I_cc_ is presented in Figure 4c, where a slope close to 1 was found from both the experimental and calculated data. This result clearly highlights that the size of the percolating CF is the key factor that governs the total resistance state of the device.^[^ ^67^ ^]^ To elaborate on the underlying origins of the recorded experimental results, a previously developed self‐consistent numerical model was used.^[^ ^68^ ^]^ According to this model, the memristive pattern strongly depends on the drift, diffusion, and thermo‐diffusion fluxes that determine the migration of Ag^+^ ions. The latter are created through oxidation and then, through their movement within the halide perovskite under the application of an external electric field, they are reduced at the inert electrode. As a result, a metallic chain that connects the two electrodes is created, which permits facile electron transport and induces the switching of the device's resistance. The filamentary conjecture stems from the independence of the LRS from the device area (Figure S12, Supporting Information). There are also experimental pieces of evidence regarding the formation of CFs in mixed organic‐inorganic halide perovskites.^[^ ^69^ ^]^ The CF is also believed to consist of metal NCs composed of Ag that are gradually created due to the very small diffusion barrier of silver atoms and the elevated solid‐solubility of Ag.^[^ ^64^, ^70^ ^]^ These effects could lead to increased material precipitation and the formation of Ag NCs when the concentration of Ag surpasses its solid‐solubility limit within the perovskite structure.^[^ ^71^ ^]^

The simulated geometry is depicted in Figures S13 and S14 (Supporting Information), while the evolution of the CF's diameter and total resistance is provided in Figures S15 and S16 (Supporting Information). The proposed model can successfully reproduce the experimental data and predict the existence of two switching modes. The central hypothesis is that the CF is self‐ruptured due to intense local Joule heating. This effect is incorporated in our model considering the impact of the thermal‐out diffusion flux.^[^ ^68^, ^72^ ^]^ The BE material plays also a key role in this direction.^[^ ^73^ ^]^ More specifically, the small thermal conductivity value of ITO (4 Wm^−1^K^−1^) negatively affects its ability to effectively dissipate the generated heat from the device's active core. According to experimental results from the literature, the melting point of Ag NCs having an average diameter of 4 nm is reduced to ≈700 K, in striking contrast with bulk Ag, which exhibits a melting point of 1233 K.^[^ ^74^ ^]^ Interestingly, such temperature values are developed during device operation and could interpret the volatile behavior of the proposed devices. The calculated map of the localized temperature distribution is displayed in Figure S17 (Supporting Information). Thus, it can be argued that the part of the CF that is in direct contact with the BE could melt down, leading to the formation of a gap region between the tip of the CF and the inert electrode. Immediately, the device will switch back to the high resistance state (HRS) without the need to alter the polarity of the applied bias. The proposed mechanism is also compatible with the ultra fast relaxation times of our prototypes, which spontaneously relax to the HRS in just nanosecond time scales (Figure S18, Supporting Information). Although the possibility of local phase transformations facilitating the migration of Ag^+^ ions away from the CF region cannot be ruled out, no such changes have been detected in the literature in similar structures.^[^ ^75^ ^]^

The statistical dispersion of the cycle‐to‐cycle (temporal) and device‐to‐device (spatial) characteristics was also examined. As far the later property is concerned, relatively small values for the coefficient of variance (σ/μ) were recorded for both the LRS and HRS of the DEAHCl sample (Figure 5d). The σ/μ ratio represents the slope of the extracted cumulative distribution functions (CDF) and should be as small as possible. Bigger variations were recorded for the reference sample, especially in the HRS, which indicate the uncontrollable rupture of the formed CF considering that the measured values exhibit a tendency to the LRS. The same pattern takes place during the investigation of the temporal dynamics of our devices during the application of either DC (Figure 5e,f) or AC (Figure 5g) measurements. In particular, in the DC scheme, a constant degradation is recorded during consecutive *I*–*V* cycling, whereas in the alternating current (AC) pulsing scheme, the same effect is manifested after the implementation of a set of ≈100 pulses, due to their lower energy. On the contrary, in the case of DEAHCl, the respective temporal properties were impeccable, suggesting that the spatial confinement effect in conjunction with the reduced surface roughness could better tune the migration of Ag^+^ ions and yield a more controllable annihilation of the CF. This effect is even more pronounced when examining the performance of our devices at different time periods. As can be seen (Figure S19, Supporting Information), the DEAHCl‐based samples remained functional after a period of 2 months, in striking contrast with the reference samples that quickly degraded after a few days. The degradation of the reference samples could be explained by considering the possible formation of AgI and AgBr layers due to the iodide‐rich perovskite structure.^[^ ^75^ ^]^ The existence of the DEAHCl film not only can effectively passivate the underlying perovskite from environmental degradation, but also slow down or hinder the above out diffusion process, yielding the development of robust memory elements.

#### 2.3.1 Role of Metal Contacts

**Figure 6:**
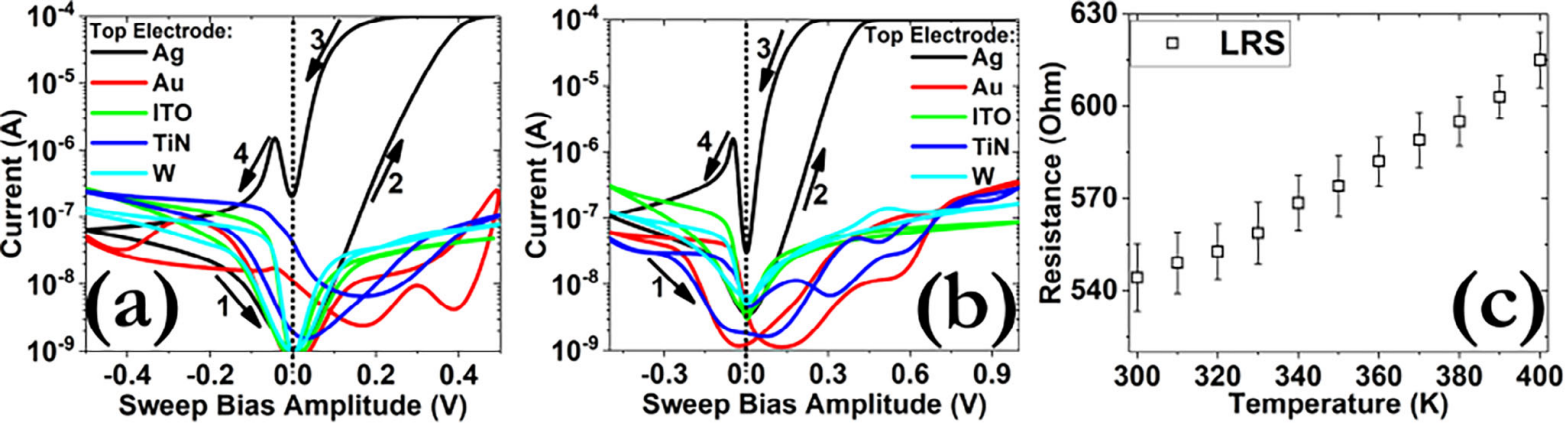
*I*–*V* hysteresis patterns using various top electrode materials under the application of a) 0.5 V and b) 1 V switching cycles. c) Dependence of the LRS of the Ag‐based sample as a function of the temperature. In all cases, the sweep rate was 10 mV s^−1^ and a constant I_cc_ of 100 µA was enforced.

Metal contacts are not merely passive electrodes but actively shape the switching characteristics and performance of perovskite‐based resistive switching devices, making their selection and optimization a key focus in device design. To assess their impact on the switching effect, various TE materials with low (ITO, TiN), intermediate (W), and high (Ag) reactivity, as well as inert electrodes (Au) were utilized. As can be observed from **Figure** **6**a,b, no switching effect was recorded when other metals than Ag were used. A forming process was also applied, leading to the permanent breakdown of the respective samples. These results are in direct line with the DFT calculations, excluding the potential contribution of other mobile species in the formation of the CF.^[^ ^76^ ^]^ The induced Schottky barrier heights could also not explain the experimentally observed results (Figure S20, Supporting Information). It is thus apparent that the utilization of reactive metals assists the filamentary conduction, while the DEAHCl layer facilitates the CF growth process. The formation of Ag‐based CFs has been also recently corroborated in the literature with the application of in‐situ visualization techniques in halide perovskite‐based memristors.^[^ ^77^ ^]^ The latter is also in direct agreement with the temperature dependence measurements of the LRS (Figure 6c), where a metallic behavior can be observed.

### 2.4 Synaptic Characteristics Under Electrical Stimulations

**Figure 7:**
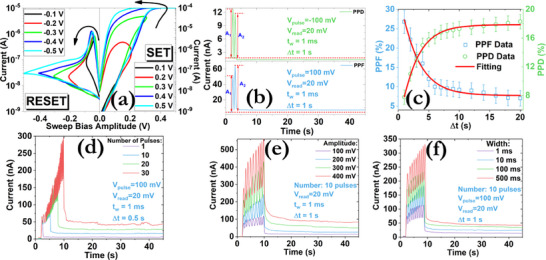
a) Continuous modulation of the conductance states under the implementation of various positive/negative voltage sweeps with a sweep rate of 10 mV s^−1^. b) EPSC responses induced by the application of two consecutive presynaptic electrical pulses with a duration (t_w_) of 1 ms and an interval (Δt) of 1 s. The parameters A_1_ and A_2_ indicate the intensities of the EPSC peaks imposed by the first and second electrical pulse, respectively. c) Distribution of the PPF and PPD indexes as a function of the pulse interval (Δt) of the electrical pulses. The red solid lines represent the fitting by an exponential function. Impact of the d) pulse number, e) pulse amplitude, and f) pulse width on the STM to LTM transition. The diverse end time of the pulse sequences in (f) can be attributed to the different width of the applied pulses. All EPSCs responses were recorded at a read‐out voltage of 20 mV and conducted on fresh devices.

Biological synapses are specialized structures in the nervous system that enable communication between neurons or between neurons and other cells. These properties make biological synapses incredibly versatile and crucial for the complex functions of the nervous system. To emulate their behavior, electrical pulses were delivered to the TE (pre‐synaptic input), and the synaptic plasticity effect was monitored on the BE (post‐synaptic output). **Figure** **7**a shows the continuous modulation of the output current by performing either consecutive positive sweeps (0→V_max_^+^→0) or negative sweeps (0→V_max_^−^→0) with increasing amplitude. This effect permits a more precise control of the synaptic strength, allowing for the emulation of better learning and memory functions in artificial neural networks.^[^ ^78^ ^]^ The dual switching modes that were previously demonstrated, were also leveraged here to reproduce the short‐term memory (STM) and long‐term memory (LTM) properties of biological synapses.^[^ ^79^ ^]^ Initially, the short‐term plasticity effects were investigated by studying the distribution of the paired‐pulse facilitation (PPF) and paired‐pulse depression (PPD) indexes (Figure 7b). A pair of pre‐synaptic electrical pulses is delivered and the effect of the timer interval (Δt) can be extracted (Figure 7c). The indexes were calculated and fitted using Equations (1) and (2), respectively:

(1) $$PPF/PPD_{ratio}=\left[\frac{\left(\left|A_{2}\right.-\left.A_{1}\right|\right)}{A_{1}}\right]\times 100\%$$

(2) $$PPF/PPD_{ratio}=C_{1}e^{\left(-\frac{t}{\tau_{1}}\right)}+C_{2}e^{\left(-\frac{t}{\tau_{2}}\right)}$$

where C1 and C2 are fitting constants and τ1 and τ2 represent the relaxation times of this effect. From our analysis, the values of τ_1_ = 2.8 s and τ_2_ = 4.7 s for PPF and τ_1_ = 2.6 s and τ_2_ = 4.1 s for PPD were extracted, which are comparable to the response time of biological synapses.^[^ ^80^ ^]^ A transition from STM to LTM was also observed by either increasing the number of the applied voltage pulses (Figure 7d) or their amplitude (Figure 7e) or their duration (Figure 7f). In all cases, the excitatory postsynaptic current (EPSC) responses were enhanced, indicating the consolidation of the various learning levels. In biological neural networks, such types of transitions involve temporary changes in synaptic strength due to recent neural activity, lasting milliseconds to minutes, and play a pivotal role in the transformation of temporary and transient information into more stable forms. The electrical power consumption of the produced EPSCs was also estimated using the following equation:

(3) $$E_{electrical}^{EPSC}=V\times I\times t$$

where V is the amplitude of the voltage pulse (100 mV), I is the current of the EPSCs (≈50 nA) and t_spike_ is their width (≈1 ms). A power consumption of 5 pJ was extracted, which is increased by a factor of ≈1.2 during the application of consecutive pre‐synaptic signals. The energy consumption of the recorded EPSCs changes was taken into account here and not just the outputs of the read pulses. The power consumption can be further reduced by decreasing the width of the applied pulses.

### 2.5 Synaptic Characteristics Under Optical Stimulations

**Figure 8:**
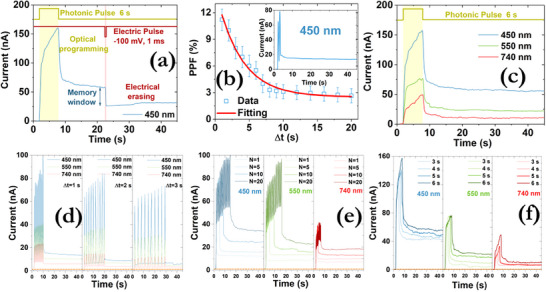
a) EPSC responses of the DEAHCl device with photonic programming and electrical erasing. The light intensity was 1.4 mW cm^−2^ at the illumination took place at a wavelength of 450 nm. b) Distribution of the PPF index as a function of the pulse interval (Δt) of the optical pulses under constant irradiation with an intensity of 1.4 mW cm^−2^ and duration of 1 ms. The red solid line denotes the fitting by an exponential function. The inset depicts the recorded EPSCs profiles with an optical pulse interval of 1 s. c) Various EPSCs responses induced by light irradiation at the wavelengths of 450, 550, and 740 nm with a pulse duration of 6 s. Dependence of the various EPSCs on d) pulse interval, e) pulse number, and f) pulse width of optical pulses with diverse wavelengths. A transition from STM to LTM can be observed. All EPSCs responses were recorded at a read‐out voltage of 20 mV.

Besides achieving synaptic excitation in a purely electrical manner, the intense interaction of halide perovskites with light,^[^ ^81^ ^]^ permits the manifestation of light‐induced synaptic functionalities. Optical synapses are considered a conceptual approach to creating communication pathways that mimic biological synapses but rely on light (optical signals) rather than electrical or chemical signals. Optical signals are less prone to heat loss compared to electrical signals, making optical synapses energy‐efficient, particularly for large‐scale systems.^[^ ^82^ ^]^ The devices were irradiated with three different light colors (blue, red, and green) from the bottom transparent electrode. As can be observed in **Figure** **8**a, the device can be optically switched to the ON state and remained at ≈58 nA, while then, the application of an electrical erase process led to the reduction of the output current to ≈29 nA. A light‐induced PPF effect was also observed under the application of consecutive light pulses (Figure 8b), extracting relaxation times τ_1_ = 1.5 s and τ_2_ = 3.7 s. The impact of the wavelength was also examined. In particular, a reduction in the wavelength from 740 nm to 450 nm yielded a temporary current increase from 50 to 155 nA, while the LTM current was also increased from 10 to 55 nA (Figure 8c). A transition from STM to LTM was also observed here for all wavelengths by either increasing the interval of the optical pulses (Figure 8d) or their number (Figure 8e) or their duration (Figure 8f). The optical energy consumption of the light‐induced pulses was calculated using the following equation:

(4) $$E_{optical}^{EPSC}=P\times W\times A$$

where P is the optical intensity (1.4 mW cm^−2^), W is the optical pulse width (1 ms), and A is the total device area (3 cm^2^). A value of 4.2 µW can be extracted, whereas an ultra low electrical power consumption was calculated for the red optical pulse (400 fJ), green (620 fJ), and blue (700 fJ), which are comparable to that of biological synapses (10–100 fJ). These experimentally reported values are among the lowest that have been reported in the literature for halide‐based perovskite memristors and the other material configurations (Table S3, Supporting Information).^[^ ^83^, ^84^ ^]^

### 2.6 Impact of Fusion Rate on the Recognition Accuracy of Reservoir Computing

Moving at the architecture level, the development of a reconfigurable synaptic array is required to allow dataflow direction with reduced energy overheads and footprint. The standard artificial neural networks (ANNs) rely on their operation to the all‐to‐all connected feed‐forward neural networks, which consist of quite a few hidden interlayers.^[^ ^85^ ^]^ Although the employment of such types of network configuration permits good advantages, in terms of elevated cognitive accuracy and learning capabilities, an enormous amount of repetitive calculations for the synaptic weight update process during the learning phase is mandatory. Therefore, the implementation of complex electrical circuitry is used in combination with high power consumption. Additionally, due to the rigid nature of the network structure, its ability to handle more than a given task is severely limited, in striking contrast with the flexibility of the human brain neural networks. To solve this issue, the concept of RC has emerged.^[^ ^86^ ^]^ Unlike the feed‐forward networks, during the training process of the RC‐based network, only the weights of the output layer are trained, whereas the rest network remains fixed. Hence, the training becomes faster and relatively simple algorithms can be used (such as linear regression), while the temporal information enclosed in sequential signals can be effectively processed. Moreover, the richness of the reservoir states can substantially improve the inference accuracy.^[^ ^87^ ^]^

**Figure 9:**
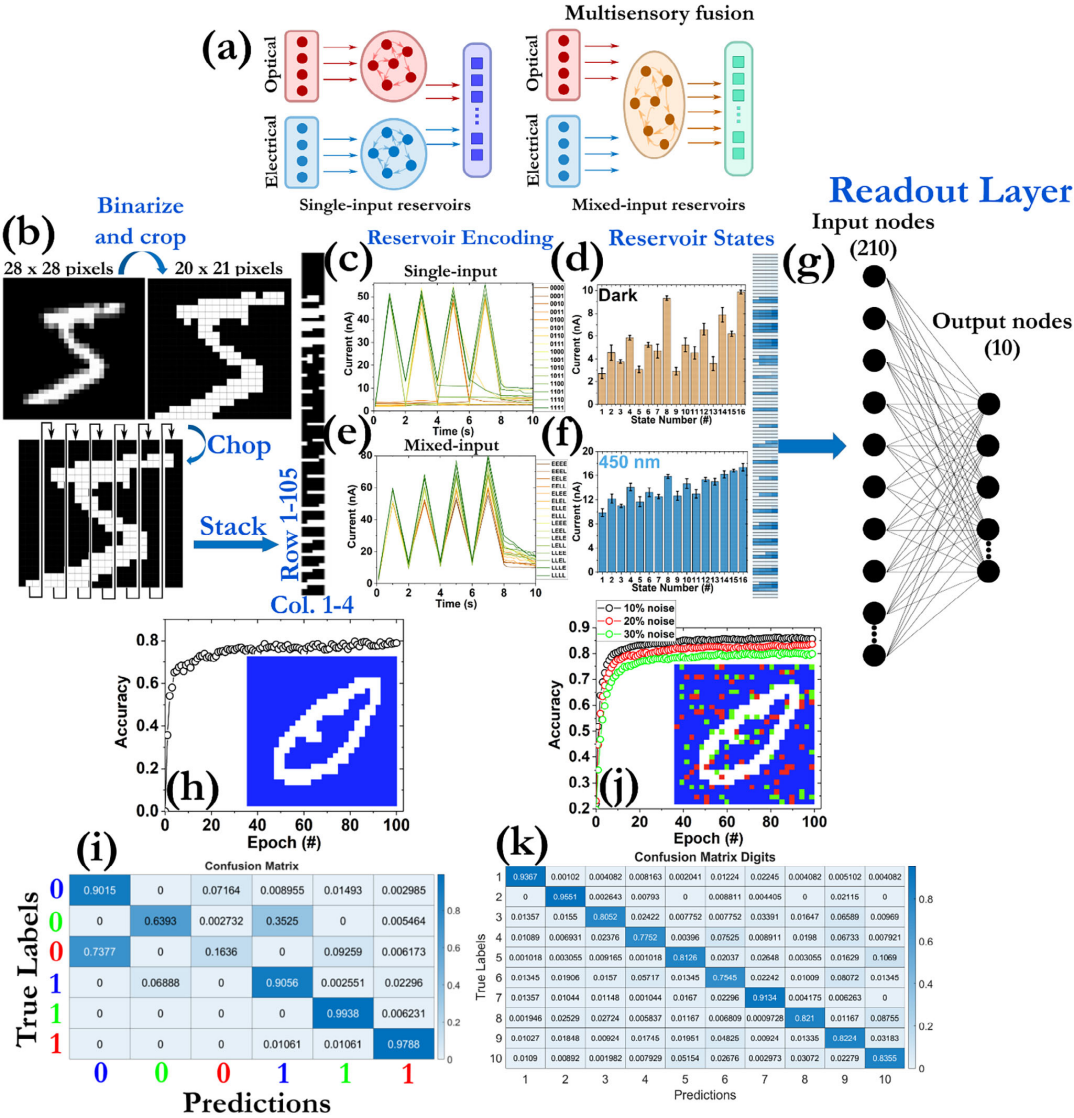
a) Schematic illustration of the single‐input and mixed‐input reservoirs. In the former case, the electrical and optical features are individually extracted by isolated reservoirs and then are used together for performing the classification tasks. In the latter case, the electrical and optical signals are fused in a mixed‐input RC system and multisensory fusion is simultaneously carried out for the classification tasks. b) Depiction of the pre‐processing procedure used for the handwritten digits of the MNIST dataset before the encoding process by the reservoir system. The initial 28 × 28 pixel images were binarized and cropped to 21 × 20 pixels. Then, they were chopped and stacked to a 105 × 4 column. The resulting patterns were translated into pulse sequences that were used as input signals of the reservoir system. c) Distribution of output current values using a 4‐bit pulse stream and applying only electrical pulses. All potential combinations of “0000” – “1111” were tested. The symbol “0” corresponds to the application of only the read‐out pulse, whereas the symbol “1” denotes the application of the electrical pulse. The width of the programming and read‐out electrical pulses was 1 ms, their interval was 1 s, while their amplitudes were 100 and 20 mV, respectively. d) Current statistics for performing pulse programming measurements under dark conditions (fully electrical). The error bar has been extracted by conducting 20 measurements for each state using different devices. The state number 1 corresponds to the combination of “0000”, the state number 2 to “0001”, the state number 3 to “0010”, and so on. e) Distribution of output current values using a 4‐bit reservoir scheme and studying the fusion of the electrical and optical pulses. All potential fused combinations of “EEEE” – “LLLL” were tested. The symbol “E” corresponds to the application of the electrical pulse, whereas the symbol “L” denotes the application of the electrical pulse. The width of the optical, electrical, and read‐out pulses was 1 ms, their interval was 1 s, while the amplitude values of the electrical and read‐out pulses were 100 and 20 mV, respectively. f) Current statistics for performing pulse programming measurements under blue light irradiation (fused with electrical). The error bar has been extracted by conducting 20 measurements for each state using different devices. The state number 1 corresponds to the combination of “EEEE”, the state number 2 to “EEEL”, the state number 3 to “EELE”, and so on. g) The outputs of the RC system were finally fed into a readout layer with a dimension of 210 × 10 to perform the classification of the classes. No hidden layers were used. The extra 105 nodes were used to take into account the negative weights, which are needed for the implementation of the backpropagation process, by applying the outputs of the reservoir with a negative polarity. h) Evolution of the training accuracy of colored MNIST dataset during the classification of 2 handwritten digits in three different single colors, having a total of 6 classes. The inset depicts the handwritten digit 0 with a blue background. i) Confusion matrix of the MNIST classification process used for testing after 100 epochs (test accuracy = 77.16%). j) Evolution of the training accuracy of colored MNIST dataset during the classification of 10 handwritten digits in a mixture of three different background colors, having a total of 10 classes. The inset depicts the handwritten digit 0 with a noise level of 10%. k) Confusion matrix of the MNIST classification process used for testing after 100 epochs (test accuracy = 84.5%).

The capability of our prototypes to tune their output currents by electrical and optical stimulation can be leveraged to develop mixed‐input reservoir systems (**Figure** **9**a).^[^ ^18^ ^]^ The underlying idea is that the application of an electrical pulse followed by an optical pulse or the opposite leads to an additional increase in the output currents, thus inducing diverse relaxation dynamics. As a result, this temporal dynamics yields different decay times compared to the case when individual electrical or light stimuli are enforced. To assess the multisensory properties of the optoelectronic RC system, the impact of the fusion scheme using a 4‐bit pulse stream was systematically examined using the MNIST dataset, which was pre‐processed (Figure 9b).^[^ ^88^ ^]^ 16 different combinations were explored by either applying fully electrical or optical signals or a mixture of them. Each input pixel is translated into one bit and then a 4‐bit encoding scheme was applied, where the sequences “0000” to “1111” refer to the binary encoding of the input electrical signals, while “EEEE” and “LLLL” were intended to denote fused electrical or fully optical pulses, respectively. In all cases, “0” refers to the OFF state of the memristor (i.e., the non‐presence of either electrical or optical pulses) and “1” to the ON state (i.e., the application of a single electrical or optical pulse). For the fused schemes, a total of 16^2^ cases were tested by examining all the potential combinations of the optical and electrical pulses (Table S4, Supporting Information). Figure 9c,e presents the distribution of the output currents under the application of 16 different inputs. As can be observed, the final current values exhibit a strong dependency on the history of the applied pulse sequence and are not only affected by the previous stimulus. This property is leveraged to encode various reservoir states, whose density is strongly related to the nonlinearity of the relaxation time of the memristor's volatile mode. As can be ascertained in Figure 9d,f, 16 distinguishable states can be achieved under dark conditions and fusion of the electrical and optical signals under blue light illumination, while similar results were recorded for the other light sources (Figures S21 and S22, Supporting Information). The accurate distinguishability of the various reservoir states is of great importance for conducting in‐memory RC computations with high recognition accuracy. Next, the various reservoir states are fed to the read‐out layer consisting of non‐volatile memristors to update their synaptic weight and perform the classification tasks.

We have to underline that both the potentiation and depression profiles can be employed to carry out RC calculations. Considering that our prototypes can be initially found in the HRS, the potentiation curves were used here. The effect of the fusion scheme on the accuracy recognition of single color images was studied considering all potential combinations for the volatile nodes of the reservoir (Figure S23, Supporting Information) and a synaptic weight modulation process with the experimentally extracted degrees of linearity (Figure S24, Supporting Information). The fused combination of “LLLE” yielded the best accuracy across the various multisensory inputs and among all light sources. The improved accuracy stems from the fact that after the application of three consecutive light pulses, the output current constantly increases at a higher rate, with respect to the fully electrical case, inducing the formation of a distinct conductance state. Although the fourth applied electrical pulse in the “LLLE” fusion mode just leads to a reduced increase rate of the current, the final reservoir state is more discernible than the others. For this reason, it was selected to process the colored MNIST dataset, which contains colored images (in Red, Green, and Blue background) of the handwritten digits (white pixels), and find the optimal fusion scheme. This particular application aimed to control the performance of optoelectronic devices when optical pulses of different wavelengths are delivered. The recognition accuracy of the system when using RGB images containing the handwritten digits 0 and 1 was 92%.

Next, the identification of the background wavelength of the images was attempted by increasing the classes from 2 to 6 (digits 0 and 1 in RGB background) and the success rate dropped slightly to close to 79% (Figure 9h,i). However, the performance deteriorated when the classes were increased to 30 for ten digits and the accuracy remained low, which is anticipated considering that full‐color recognition is not a time‐dependent quantity. Interestingly, an accuracy of 88.6% was calculated in recognizing 10 classes of handwritten RGB digits with different single color backgrounds (Figure S25, Supporting Information). Finally, the capability of the proposed system to carry out the latter task with the introduction of noise in the background of the colorful images was studied. The added noise of each color image was different from the color background (i.e. if the background was blue, the pixels got red or green values) and followed a uniform distribution. Figure 9j,k show that the recognition accuracy can be as high as 83.5% even for the case of 20% noise, highlighting the unique capabilities of the proposed RC system to accurately recognize various patterns in blurred environments (Figure S26, Supporting Information).

### 2.7 Multimodal In‐Sensor Computing for Real‐Time Vision and Audio Monitoring

**Figure 10:**
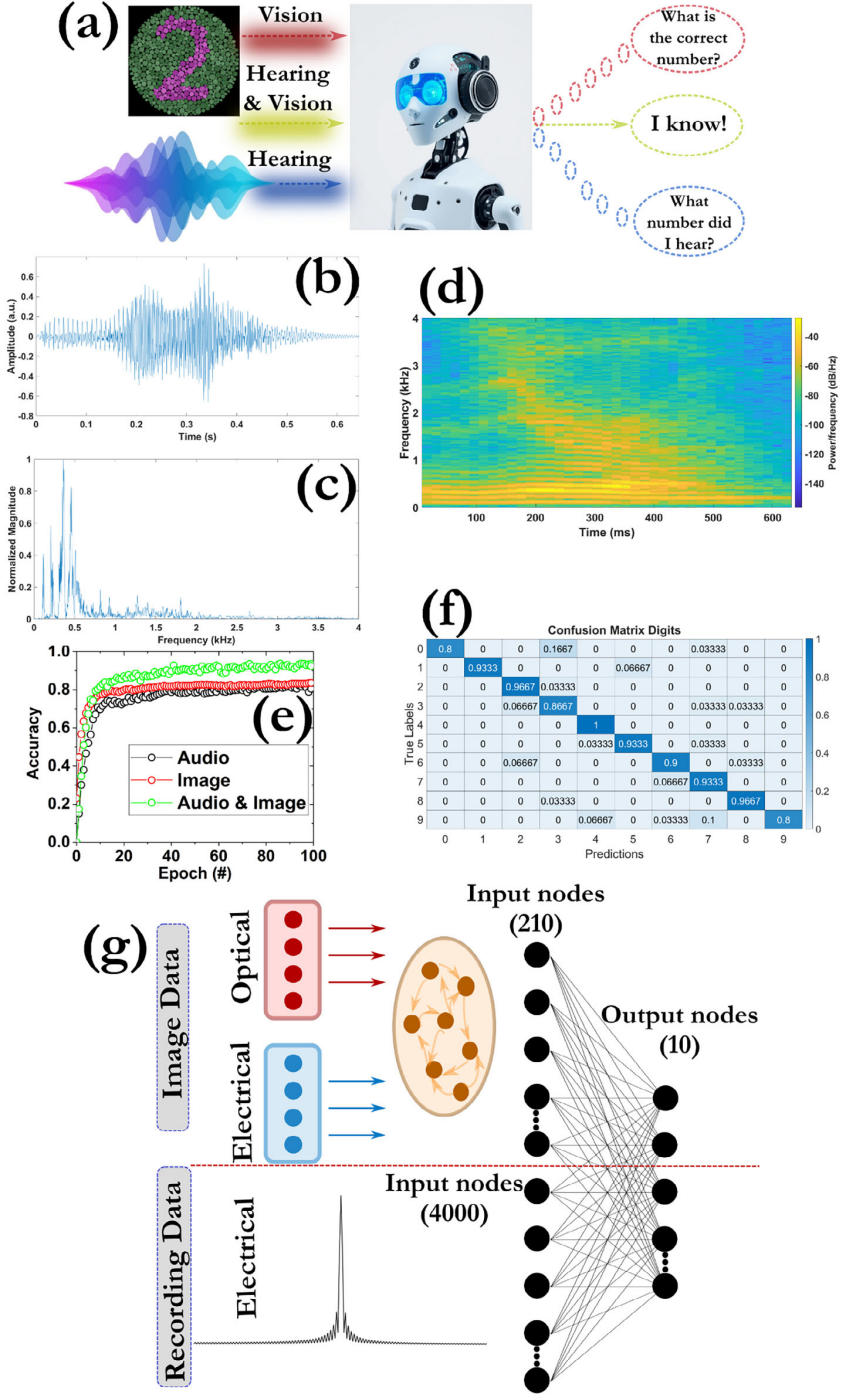
a) Emulation of the visual‐audio information process. b) Distribution of the input signal and its c) Fourier transformation. d) Speech spectrogram of the spoken digits dataset indicating the frequency domain distribution of the audio signals at various time moments. A color bar is also included that represents the power spectral density (PSD) in decibels (dB), indicating the intensity of each frequency component at a given time. This helps interpret the strength of signals across different frequencies over time. e) Evolution of the training accuracy during single (classification of colored MNIST and spoken digits datasets) or multimodal processing in recognizing 10 classes of digits. f) Confusion matrix of the multimodal classification process used for testing after 100 epochs (accuracy = 91.2%). g) Schematic illustration of the overall multimodal processing framework. The image and electrical recording data, after being processed through reservoir computing and Fourier transformation, respectively, are simultaneously input to the output layer comprising 4210 × 10 nodes. No hidden layers were used.

Recognizing patterns from both audio and visual signals simultaneously (multimodal recognition) is crucial for applications such as speech recognition in noisy environments, enhanced environment exploration or even adaptive robotics. By mimicking the efficiency and adaptability of biological systems, neuromorphic vision and hearing represent a leap forward in sensory technology, with transformative potential across industries.^[^ ^88^, ^89^ ^]^ Neuromorphic vision and hearing systems can process data asynchronously, only responding to dynamic changes in the environment (event‐driven processing), reducing unnecessary computations and power usage. Thus, less data are generated, significantly simplifying downstream processing. Memristors facilitate the integration of these signals by processing and linking them within the same network, potentially achieving cross‐modal processing (**Figure** **10**a). On the contrary, conventional systems often require constant frame‐by‐frame processing for machine vision and the entire sound spectrum is analyzed for voice recognition (even in the absence of meaningful audio signals), which is energy‐intensive. As a result, latency could be introduced due to the need for frame capturing and sequential processing. They also may suffer from motion blur or limited frame rates in fast‐moving scenarios or struggle in noisy environments, requiring extensive signal processing to filter out background noise. Conventional systems generate dense datasets (full image frames), requiring more storage and processing power.

As a proof of concept, the dynamic perception of humans in recognizing digits from both visual and audio information was emulated. First, the Free Spoken Digit Dataset (FSDD) was used, which contains audio/speech recordings of spoken digits (0‐9 digits) from three speakers at 8 kHz. The speech input recordings (Figure 10b) were processed using a 4000‐point Fourier transform to analyze the frequency components (Figure 10c), and only 2000 points of the normalized magnitude were handled, because they correspond to the positive frequency components. The speech spectrogram of a zero‐sample is depicted in Figure 10d to offer a visual representation of the frequency content of a signal as it changes over time. Then, the generated values were passed to a memristive read‐out layer (with dimensions 4000 x 10) to carry out the final recognition. The final accuracy on the test set was moderately high at 76% (Figure S27, Supporting Information). Finally, we checked the ability of the devices and by extension the system to manage multimodal data, by combining the noisy RGB MNIST images (20% added noise) and the FSDD audio recordings. The noisy images were passed through an optoelectronic memristive reservoir, while the audio recordings were Fourier transformed through the electrical memristive crossbar. The transformed values of the audio‐visual signals were applied to a common read‐out layer together with a bias term, which had a size of 4212 x 10 (2000 x 10 for the Fourier transformed audio signals, 105 x 10 for the non‐linear transformation of the reservoir visual images, and one word line for the bias term, the final 4212 word lines are used for obtaining both positive and negative weights).

Figure 10e illustrates the training accuracy over epochs, comparing the multimodal system to cases where input data was sourced from individual modalities. After 100 epochs, the recognition accuracy of the vision and audio patterns was lower than 83%, suggesting the difficulties in recognizing different patterns using a single mode information processing protocol. Considering that the proposed device configuration can respond to a variety of electrical (audio dataset) and optical (vision dataset) signals, a multimodal recognition using the reservoir and neural networks, while the outputs were fused and analyzed through a readout crossbar layer. The training accuracy of the multimodal system greatly increased to over 93.1%, significantly surpassing the accuracy of the other curves, highlighting the improved performance of the multimodal computing scheme that stems from the reconfigurability of the halide‐based memory devices. The confusion matrix of the multisensory inputs is highlighted in Figure 10f stating a test accuracy of 91%.

## 3 Conclusion

In this work, a neuromorphic halide perovskite device with multimodal memory, sensing, and in‐situ processing properties was developed and systematically examined. The devices responded to various electrical and optical stimuli at various time scales, which were leveraged to emulate reconfigurable neuromorphic properties. In particular, the volatile characteristics and the tunable relaxation times were used as reservoir nodes to process and analyze temporal data by introducing nonlinear transformations. On the contrary, the non‐volatile characteristics were employed to perform artificial synaptic functionalities and pattern classification. The RC systems harness the collective dynamics of these nodes to efficiently transform and analyze data, offering both simplicity and scalability for a wide range of tasks. The colored MNIST database was used as a standard test, yielding a recognition accuracy of ≈79% in recognizing multicolor images. The impact of the fusion rate of the electrical and optical signals was also investigated and the optimal scheme was selected, based on recognition accuracy rate and power consumption. Multimodal recognition properties, comparable to the human brain, were also demonstrated by performing vision and audio monitoring in real time conditions, yielding a training accuracy rate of 91%. In parallel, the properties of the DEAHCl interface with the CsFAMA film were thoroughly studied by carrying out DFT calculations, shedding light on the formation origins of the LD layer and its impact on the stability of the Ag‐based CF. The crucial role of the latter was highlighted by examining the influence of the TE on the manifestation of the resistive switching effect. Valuable insights regarding the formation of percolating CFs were also extracted by the C‐AFM measurements. Our device's ability to integrate and interpret information from multiple sensory modalities (vision, hearing) to create a unified perception of the environment makes it highly attractive for the future design and development of low‐power intelligent systems with advanced cognitive capabilities.

## 4 Experimental Section

### Materials

Lead iodide (PbI_2_, 99.99%) was supplied by TCI EUROPE N.V., Formamidinium iodide (FAI, 99.99%), Cesium iodide (CsI, 99.99%), Methylammonium Chloride (MACl, 99.99%) and Methylammonium Bromide (MABr, 99.99%) was acquired from Greatcell Solar Materials. N,N‐dimethylformamide (DMF, 99.8%), dimethylsulfoxide (DMSO, 99.7%), isopropyl alcohol (IPA, 99.5%), chlorobenzene (CB, 99.5%) and acetonitrile (99.9%) were purchased from Acros Organics B.V.B.A. 2‐Diethylaminoethanethiol hydrochloride (DEAHCl) was purchased from Sigma–Aldrich. The materials were used without further purification.

### Device Fabrication and Optoelectronic Measurements

Indium‐doped tin oxide (ITO) substrates were used. ITO was sputtered on m‐glass were room temperature using a high purity ITO target (purity 99.99%). The thickness of the ITO layer was 40 nm and the deposition took place at a base pressure of 4 x 10^−3^ mbar delivering a total power of 150 W. Then, the substrates were cleaned by sonication washing with detergent (Helmanex III 2% in deionized water), deionized water, acetone, and isopropanol for 15 min. Before perovskite deposition, the cleaned ITO substrates were treated with ultraviolet ozone for 30 min and then were transferred immediately into N_2_ glove box. The precursor solution of perovskite was prepared by adding 1.6M PbI_2_, 1.39M FAI, 0.08M MABr and 22 mg MACl in 1 ml of DMF‐DMSO (4:1, v/v) mixture, and 47 µL of CsI (1.5 M in DMSO) was added and stirred for 2 hours. The role of MACl was in assisting the crystallization process (not directly incorporated into the perovskite crystal structure). 60 µL of the solution were spin coated on ITO substrates at 2000 rpm for 10 s and 6000 rpm for 25 s. At 10th second of the 2nd step, 150 µL IPA were deposited on the spinning substrate. The resulted films were immediately annealed at 100 °C for 10 min in glovebox and then at 150 °C for 15 min in air ≈25‐30% relative humidity. After the perovskite annealing process, the films were transferred into a glovebox and then 60 µL of DEAHCl solution (1 mg mL^−1^ in isopropyl alcohol) were spin‐coated dynamically on the surface of the film at 3000 rpm for 30 s and annealed for 1 min at 100 ^°^C. The total thickness of the CsFAMA film was ≈600 nm, while the thickness of the DEAHCl was ≈5 nm. Finally, to obtain the complete devices, 40 nm of patterned Ag electrodes through a shadow mask were sputtered under 10^−3^ mbar pressure using a high purity silver target (purity 99.99%) with a total power of 200 W. The electrical measurements were performed at room temperature with the Keithley 4200 semiconductor parameter analyzer (4200‐SCS) at the SUSS MicroTec probe station. Pulse‐bias measurements were performed through the Keithley 4200 pulse measurement unit along with the Keithley 4225 remote sensing amplifiers. The triggering of the power LED 1 W array was conducted using the HP 8116A Pulse Generator. The intensity of the light beam was measured using the Gentec‐EO laser power and energy meter with display (model: MAESTRO).

### Materials Characterization

The surface morphologies of the perovskite films were prompted with SEM imaging using a JEOL 7401f Field Emission SEM and AFM using a Solver Nano AFM in Contact Mode with a CSG30 probe. The hydrophilicity of the perovskite films, with and without DEAHCl treatment, was examined through a contact angle goniometer (Ossila). The UV–vis transmittance spectra of the perovskite films were recorded with a Carry 60 Agilent UV–vis spectrometer. Steady‐state photoluminescence spectra were recorded on a Horiba Fluoromax+ spectrofluorometer, equipped with a 150‐W Xe lamp as the excitation source and a Hamamatsu R13456 PMT (185‐980 nm nominal) as the emission detector. The crystal structure of the perovskite films was analyzed by the recorded XRD patterns, which were obtained with a SmartLab Rigaku diffractometer. The XPS data were collected with a PHOIBOS 100 (SPECS) hemispherical analyzer using Mg Ka X‐ray source with photon energy 1253.64 eV. Voigt functions were used for the fitting analysis after standard Shirley background subtraction. The work function and valence band maximum values for the perovskite films were extracted by conducting UPS measurements using a Helium excitation source with He I radiation at 21.22 eV.

### C‐AFM Measurements

In this study, a Park NX‐Hivac from Park Systems was used, operating in a high vacuum atmosphere (10^−6^mbar). For the C‐AFM measurements, a RMN‐25Pt300b was used from Rocky Mountain Nanotechnology (nominal R_TIP_ = 20 nm and k = 18 N m^−1^). The tip and cantilever were made from solid Pt and the C‐AFM measurements were performed in contact mode operation using a VECA DLPCA‐200 current amplifier. This tip was mechanically more stable than the metal‐coated ones, allowing high‐resolution topography and conductive maps in films with high topographic features. The whole active area was scanned by applying a bias from the substrate at 1 V, so that the CF properties were not modified.

### Laser Ablation Experiments

An Nd:Yag laser, featuring a central wavelength of 532 nm, a pulse duration of 20 ns, 20 W, maximum output power and a 500 kHz maximum repetition rate, was employed for the ablation of the silver electrodes, combined with a galvanometric scanner enabling an X‐Y direction sample scanning. The optimal Ag layer removal was achieved with a 60 kHz repetition rate, 35 micrometer laser spot size, 0.1 m s^−1^ galvo scanning speed and a 35 mW laser power.

### DFT Calculations

The results were obtained with the Density Functional Theory (DFT) code Vienna Ab Initio Simulation Package (VASP)^[^ ^90^ ^]^ with a plane‐wave basis (and an energy cutoff of 500 eV), projector augmented waves (PAW)^[^ ^91^ ^]^ and the generalized gradient approximation (GGA) Perdew‐Burke‐Ernzerhof exchange‐correlation (xc) functional.^[^ ^92^ ^]^ The non‐bonding van der Waals interactions were taken into account within the so‐called DFT‐D3 scheme.^[^ ^93^ ^]^ Structures were rendered with the software VESTA.^[^ ^94^ ^]^ The calculations of the barriers employed the Nudged Elastic Band (NEB) method^[^ ^95^ ^]^ with 10 intermediate so‐called images. For the studies on the bulk perovskite, cubic FAPbI_3_ supercells were used with a length of 12.72 Å in each direction. The results were based on supercells with one Br atom out of a total of 24 halogen sites, while in certain calculations, one FA cation was replaced by a Cs cation in order to probe the role of the latter. For the PbI_2_ calculations, supercells were used with 24 Pb and 48 I atoms.

### Artificial Neural Network and Reservoir Computing Simulations

The RC simulations were performed considering a 4‐bit pulse stream input. The original 28 x 28 input image was cropped to 21 x 20 pixels and then was transformed into a 105 x 4 image. The currents from the four bit lines were selected and transformed into normalized voltage vectors. At the same time, the input audio signal recordings were Fourier transformed using a Nmax2000 memristive crossbar, where Nmax was the maximum dimension of the input signals. Therefore, the final read‐out layer has dimensions of 2105x10 and it apply an additional bias term of 1V. If it account for the desired negative weights the read‐out layer should have 4212 word lines and 10 bit lines. Regarding the training and test data, the audio recordings of 3 speakers were used with 50 samples per digit, resulting in a total of 1500 samples. From the total samples, 1200 were used for training and 300 for testing. As the visual data was much more each training epoch 1200 image data were chosen randomly and 300 test images for the test forward pass. As the visual data was much more each training epoch 1200 image data were chosen randomly and 300 test images for the final test forward pass. The multimodal RC system was trained within 100 training epochs, where each training epoch the data were split into 48 batches and 25 examples per batch. To obtain the estimated class the Bayesian probabilities of the output currents were calculated. To estimate the total power consumption during the forward pass, the power consumption due to the pulse streams in the volatile optoelectronic memristors and the consumption in the non‐volatile crossbar. 105 different pulse series of four pulses were applied to the reservoir, where it considered that each of the first three corresponds to a different wavelength and the last one was electrical. Therefore, taking into account the mentioned energies, it can calculate that it have 105*(0.4+0.65+0.5+5) pJ with a pulse duration of 1 ms resulting in 690 nW. The power consumption of the optoelectronic part of reservoir computing was among the lowest values reported in the literature (Table S3, Supporting Information). Regarding the memristive crossbar, the consumption through the voltage vector and the matrix G as V^2^/R can be directly calculated. Taking the sum of all 4212 elements, a value of 37 mW was extracted. It was visible that more power was consumed in the crossbar array as it contains more elements, while at the same time, it was programmed electrically. The calculated power consumptions were strongly connected with the selected application were have to underline and thus a direct comparison with other works in the literature was not possible.

### Statistical Analysis

All measurements were performed on at least one hundred independently fabricated devices to ensure reproducibility. Raw electrical data were pre‐processed by removing outliers beyond three standard deviations from the mean and normalized where appropriate to enable direct comparison across samples. The results were presented as mean ± standard deviation (SD) unless stated otherwise. The sample size (*n*) for each analysis is provided in the corresponding figure captions. Statistical comparisons between datasets were performed using a two‐tailed Student's *t*‐test with a significance level of *α* = 0.05. In cases involving multiple comparisons, a one‐way ANOVA followed by Tukey's post‐hoc test was applied to assess statistical significance. All statistical analyses were conducted using OriginPro 2017 software. For AFM roughness analysis, data from 256 line scans per image were averaged over 2–3 distinct areas per sample and at least three independent samples.

### Ethical Statement

The data regarding optical and audio signal recognition were from open source databases of MNIST and FSDD, respectively. In particular, the audio samples used in this study were obtained from the publicly available Free Spoken Digit Dataset (FSDD), developed by Zohar Jackson^[^ ^96^ ^]^ and distributed under the Creative Commons Attribution‐ShareAlike 4.0 International (CC BY‐SA 4.0) license. The dataset allows free use, sharing, and adaptation for research purposes, provided appropriate credit was given. Accordingly, it acknowledge the original creator, Zohar Jackson, and the source of the dataset. No additional permissions were required beyond compliance with the CC BY‐SA 4.0 terms. All experiments in this work do not involve related ethics problems.

## Conflict of Interest

The authors declare no conflict of interest.

## Supporting information



Supporting Information

## Data Availability

The data that support the findings of this study are available from the corresponding author upon reasonable request.

## References

[1] B. Li , F. Xia , B. Du , S. Zhang , L. Xu , Q. Su , D. Zhang , J. Yang , Adv. Sci. 2024, 11, 2310263. 10.1002/advs.202310263 PMC11187899 38647431

[2] H. S. P. Wong , S. Salahuddin , Nat. Nanotechnol. 2015, 10, 191. 25740127 10.1038/nnano.2015.29

[3] W. Shi , J. Cao , Q. Zhang , Y. Li , L. Xu , IEEE Internet Things J. 2016, 3, 637.

[4] H. Seok , D. Lee , S. Son , H. Choi , G. Kim , T. Kim , Adv. Electron. Mater. 2024, 2300839.

[5] X. Ji , X. Zhao , M. C. Tan , R. Zhao , Adv. Intell. Syst. 2020, 2, 1900118.

[6] Z. Wang , S. Joshi , S. E. Savel'ev , H. Jiang , R. Midya , P. Lin , M. Hu , N. Ge , J. P. Strachan , Z. Li , Q. Wu , M. Barnell , G.‐L. Li , H. L. Xin , R. S. Williams , Q. Xia , J. J. Yang , Nat. Mater. 2017, 16, 101. 27669052 10.1038/nmat4756

[7] Md. A. Rahman , S. Walia , S. Naznee , M. Taha , S. Nirantar , F. Rahman , M. Bhaskaran , S. Sriram , Adv. Intell. Syst. 2020, 2, 2000094.

[8] S. Ambrogio , P. Narayanan , H. Tsai , R. M. Shelby , I. Boybat , C. di Nolfo , S. Sidler , M. Giordano , M. Bodini , N. C. P. Farinha , B. Killeen , C. Cheng , Y. Jaoudi , G. W. Burr , Nature 2018, 558, 60. 29875487 10.1038/s41586-018-0180-5

[9] P. Yao , H. Wu , B. Gao , J. Tang , Q. Zhang , W. Zhang , J. J. Yang , H. Qian , Nature 2020, 577, 641. 31996818 10.1038/s41586-020-1942-4

[10] S. Wang , S. Gao , C. Tang , E. Occhipinti , C. Li , S. Wang , J. Wang , H. Zhao , G. Hu , A. Nathan , R. Dahiya , L. G. Occhipinti , Nat. Commun. 2024, 15, 4671. 38821961 10.1038/s41467-024-48908-8 PMC11143376

[11] X. Luo , C. Chen , Z. He , M. Wang , K. Pan , X. Dong , Z. Li , B. Liu , Z. Zhang , Y. Wu , C. Ban , R. Chen , D. Zhang , K. Wang , Q. Wang , J. Li , G. Lu , J. Liu , Z. Liu , W. Huang , Nat. Commun. 2024, 14, 3086. 10.1038/s41467-024-47374-6 PMC11006927 38600063

[12] G. Feng , X. Zhang , B. Tian , C. Duan , InfoMat 2023, 5, 12473.

[13] J. Zhu , X. Zhang , R. Wang , M. Wang , P. Chen , L. Cheng , Z. Wu , Y. Wang , Q. Liu , M. Liu , Adv. Mater. 2022, 34, 2200481. 10.1002/adma.202200481 35429020

[14] J. Yu , X. Yang , G. Gao , Y. Xiong , Y. Wang , J. Han , Y. Chen , H. Zhang , Q. Sun , Z. L. Wang , Sci. Adv. 2021, 7, abd9117. 10.1126/sciadv.abd9117 PMC7968845 33731346

[15] H. Tan , Y. Zhou , Q. Tao , J. Rosen , S. van Dijken , Nat. Commun. 2021, 12, 1120. 33602925 10.1038/s41467-021-21404-z PMC7893014

[16] Y. Wu , W. Deng , K. Li , X. Wang , B. Liu , J. Li , Z. Chen , Y. Zhang , Adv. Mater. 2024, 36, 2312094. 10.1002/adma.202312094 38320173

[17] J. Lao , M. Yan , B. Tian , C. Jiang , C. Luo , Z. Xie , Q. Zhu , Z. Bao , N. Zhong , X. Tang , L. Sun , G. Wu , J. Wang , H. Peng , J. Chu , C. Duan , Adv. Sci. 2022, 9, 2106092. 10.1002/advs.202106092 PMC9130913 35285175

[18] K. Liu , T. Zhang , B. Dang , L. Bao , L. Xu , C. Cheng , Z. Yang , R. Huang , Y. Yang , Nat. Electron. 2022, 5, 761.

[19] P. Li , M. Zhang , Q. Zhou , Q. Zhang , D. Xie , G. Li , Z. Liu , Z. Wang , E. Guo , M. He , C. Wang , L. Gu , G. Yang , K. Jin , C. Ge , Nat. Commun. 2024, 15, 3257. 38627413 10.1038/s41467-024-47580-2 PMC11021444

[20] S. Wang , X. Chen , C. Zhao , Y. Kong , B. Lin , Y. Wu , Z. Bi , Z. Xuan , T. Li , Y. Li , W. Zhang , E. Ma , Z. Wang , W. Ma , Nat. Electron. 2023, 6, 281.

[21] M. Pei , Y. Zhu , S. Liu , H. Cui , Y. Li , Y. Yan , Y. Li , C. Wan , Q. Wan , Adv. Mater. 2023, 35, 2305609. 10.1002/adma.202305609 37572299

[22] Y. Gong , X. Xing , X. Wang , R. Duan , S.‐T. Han , B. K. Tay , Adv. Funct. Mater. 2024, 34, 2406547.

[23] J. Zha , Y. Xia , S. Shi , H. Huang , S. Li , C. Qian , H. Wang , P. Yang , Z. Zhang , Y. Meng , W. Wang , Z. Yang , H. Yu , J. C. Ho , Z. Wang , C. Tan , Adv. Mater. 2024, 36, 2308502. 10.1002/adma.202308502 37862005

[24] H. Fang , S. Ma , J. Wang , L. Zhao , F. Nie , X. Ma , W. Lό , S. Yan , L. Zheng , Adv. Funct. Mater. 2024, 34, 2409045.

[25] Y. Geng , J. Guo , H. Wang , S. D. Ling , Z. Chen , S. Chen , J. Xu , Small 2022, 18, 2200740. 10.1002/smll.202200740 35398978

[26] E. Rezaee , D. Kutsarov , B. Li , J. Bi , S. R. P. Silva , Sci. Rep. 2022, 12, 7411. 35523822 10.1038/s41598-022-10790-z PMC9076914

[27] Y. Fang , S. Zhai , L. Chu , J. Zhong , ACS Appl. Mater. Interfaces 2021, 13, 17141. 33844908 10.1021/acsami.1c03433

[28] K. Sakhatskyi , R. A. John , A. Guerrero , S. Tsarev , S. Sabisch , T. Das , G. J. Matt , S. Yakunin , I. Cherniukh , M. Kotyrba , Y. Berezovska , M. I. Bodnarchuk , S. Chakraborty , J. Bisquert , M. V. Kovalenko , ACS Energy Lett. 2022, 7, 3401. 36277137 10.1021/acsenergylett.2c01663 PMC9578653

[29] W. Peng , C. Aranda , O. M. Bakr , G. Garcia‐Belmonte , J. Bisquert , A. Guerrero , ACS Energy Lett. 2018, 3, 1477.

[30] I. Chao , Y. T. Yang , M. H. Yu , C. H. Chen , C. H. Liao , B. H. Lin , I. Ni , W. C. Chen , A. W. Y. Ho‐Baillie , C. C. Chueh , Small 2023, 19, 2207734. 10.1002/smll.202207734 36794296

[31] Y. Sun , M. Tai , C. Song , Z. Wang , J. Yin , F. Li , H. Wu , F. Zeng , H. Lin , J. Phys. Chem. C 2018, 122, 6431.

[32] L. Guo , H. Sun , L. Min , M. Wang , F. Cao , L. Li , Adv. Mater. 2024, 36, 2402253. 10.1002/adma.202402253 38553842

[33] G. S. H. Thien , M. Ab Rahman , B. K. Yap , N. M. L. Tan , Z. He , P.‐L. Low , N. K. Devaraj , A. F. Ahmad Osman , Y.‐K. Sin , K.‐Y. Chan , ACS Omega 2022, 7, 39472. 36385870 10.1021/acsomega.2c03206 PMC9648113

[34] S. Satapathi , K. Raj , Yukta , M. A. Afroz , Phys. Rev. Appl. 2022, 18, 017001.

[35] J. Carlos , P. Martνnez , M. Berruet , C. Gonzales , S. Salehpour , A. Bahari , B. Arredondo , A. Guerrero , Adv. Funct. Mater. 2023, 33, 2305211.

[36] T. Wan , B. Qu , H. Du , X. Lin , Q. Lin , D. W. Wang , C. Cazorla , S. Li , S. Liu , D. Chu , J. Colloid Interface Sci. 2018, 512, 767. 29112927 10.1016/j.jcis.2017.10.113

[37] C. Gonzales , A. Guerrero , J. Phys. Chem. Lett. 2023, 14, 1395. 36738280 10.1021/acs.jpclett.2c03669 PMC9940207

[38] N.‐K. Pendyala , C. Gonzales , A. Guerrero , Small Struct. 2024, 6, 2400380.

[39] F. Xia , Y. Xu , B. Li , W. Hui , S. Zhang , L. Zhu , Y. Xia , Y. Chen , W. Huang , ACS Appl. Mater. Interfaces 2020, 12, 15439. 32148014 10.1021/acsami.9b22732

[40] S. Lee , H. Kim , D. H. Kim , W. B. Kim , J. M. Lee , J. Choi , H. Shin , G. S. Han , H. W. Jang , H. S. Jung , ACS Appl. Mater. Interfaces 2020, 12, 17039. 32174107 10.1021/acsami.9b22918

[41] X. Pan , X. Chen , J. Duan , Y. Long , Y. Wu , J. Tang , G. Ma , J. Zhang , H. Wang , ACS Appl. Electron. Mater. 2023, 5, 6908.

[42] M. Loizos , K. Rogdakis , W. Luo , P. Zimmermann , A. Hinderhofer , J. Lukic΄ , M. Tountas , F. Schreiber , J. V. Milic΄ , E. Kymakis , Nanoscale Horiz. 2024, 9, 1146. 38767026 10.1039/d4nh00104d PMC11195346

[43] M. Vasilopoulou , A. R. bin , M. Yusoff , Y. Chai , M.‐A. Kourtis , T. Matsushima , N. Gasparini , R. Du , F. Gao , M. K. Nazeeruddin , T. D. Anthopoulos , Y.‐Y. Noh , Nat. Electron. 2023, 6, 949.

[44] G. Ding , B. Yang , R.‐S. Chen , W.‐A. Mo , K. Zhou , Y. Liu , G. Shang , Y. Zhai , S.‐T. Han , Y. Zhou , Small 2021, 17, 2103175. 10.1002/smll.202103175 34528382

[45] R. Szostak , J. C. Silva , S. H. Turren‐Cruz , M. M. Soares , R. O. Freitas , A. Hagfeldt , H. C. N. Tolentino , A. F. Nogueira , Sci. Adv. 2019, 5, 2. 10.1126/sciadv.aaw6619 PMC6814396 31692661

[46] G. Lucarelli , F. De Rossi , B. Taheri , T. M. Brown , F. Brunetti , Energy Technol. 2022, 10, 2200314.

[47] Z. Hu , Q. An , H. Xiang , L. Aigouy , B. Sun , Y. Vaynzof , Z. Chen , ACS Appl. Mater. Interfaces 2020, 12, 54824. 33226765 10.1021/acsami.0c17258

[48] T. Bu , J. Li , W. Huang , W. Mao , F. Zheng , P. Bi , X. Hao , J. Zhong , Y. B. Cheng , F. Huang , J. Mater. Chem. A 2019, 7, 6793.

[49] B.‐W Park , N. Kedem , M. Kulbak , D. Y. Lee , W. S. Yang , N. J. Jeon , J. Seo , G. Kim , K. J. Kim , T. J. Shin , G. Hodes , D. Cahen , S. I. Seok , Nat. Commun. 2018, 9, 3301. 30120225 10.1038/s41467-018-05583-w PMC6098034

[50] Z. Wu , M. Jiang , Z. Liu , A. Jamshaid , L. K. Ono , Y. Qi , Adv. Energy Mater. 2020, 10, 1903696.

[51] Y. Zhou , H. Zhong , J. Han , M. Tai , X. Yin , M. Zhang , Z. Wu , H. Lin , J. Mater. Chem. A 2019, 7, 26334.

[52] Z. Hawash , S. R. Raga , D. Y. Son , L. K. Ono , N. G. Park , Y. Qi , J. Phys. Chem. Lett. 2017, 8, 3947. 28767259 10.1021/acs.jpclett.7b01508

[53] R. Garai , R. K. Gupta , M. Hossain , P. K. Iyer , J. Mater. Chem. A 2021, 9, 26069.

[54] J. J. Yoo , S. Wieghold , M. C. Sponseller , M. R. Chua , S. N. Bertram , N. T. P. Hartono , J. S. Tresback , E. C. Hansen , J.‐P. Correa‐Baena , V. Bulović , T. Buonassisi , S. S. Shin , M. G. Bawendi , Energy Environ. Sci. 2019, 12, 2192.

[55] M. A. Mahmud , T. Duong , Y. Yin , H. T. Pham , D. Walter , J. Peng , Y. Wu , L. Li , H. Shen , N. Wu , N. Mozaffari , G. Andersson , K. R. Catchpole , K. J. Weber , T. P. White , Adv. Funct. Mater. 2020, 30, 1907962.

[56] Y. Cho , A. M. Soufiani , J. S. Yun , J. Kim , D. S. Lee , J. Seidel , X. Deng , M. A. Green , S. Huang , A. W. Y. Ho‐Baillie , Adv. Energy Mater. 2018, 8, 1703392.

[57] Q. Jiang , Y. Zhao , X. Zhang , X. Yang , Y. Chen , Z. Chu , Q. Ye , X. Li , Z. Yin , J. You , Nat. Photonics 2019, 137, 460.

[58] D. Xin , S. Tie , X. Zheng , J. Zhu , W. Zhang , J. Energy Chem. 2020, 46, 173.

[59] J. Lu , X. Lin , X. Jiao , T. Gengenbach , A. D. Scully , L. Jiang , B. Tan , J. Sun , B. Li , N. Pai , U. Bach , A. N. Simonov , Y. B. Cheng , Energy Environ. Sci. 2018, 11, 1880.

[60] H. Xu , G. Liu , X. Xu , S. Xu , L. Zhang , X. Chen , H. Zheng , X. Pan , Sol. RRL 2020, 4, 2000647.

[61] D. Sakellaropoulos , P. Bousoulas , C. Papakonstantinopoulos , S. Kitsios , D. Tsoukalas , IEEE Electron Device Lett. 2020, 41, 1013. 10.1088/1361-6528/aba3a1 32634787

[62] S. Kitsios , P. Bousoulas , D. Spithouris , M. Kainourgiaki , M. Tsigkourakos , P. Chatzopoulou , G. P. Dimitrakopulos , P. Komninou , D. Tsoukalas , ACS Appl. Electron. Mater. 2022, 4, 2869.

[63] G. Kleitsiotis , P. Bousoulas , S. D. Mantas , C. Tsioustas , I. A. Fyrigos , G. Sirakoulis , D. Tsoukalas , IEEE Trans. Electron Devices 2024, 71, 5313.

[64] L. Tang , Y. Huang , C. Wang , Z. Zhao , Y. Yang , J. Bian , H. Wu , Z. Zhang , D. W. Zhang , Adv. Electron. Mater. 2021, 8, 2100771.

[65] Y. Huang , L. Tang , C. Wang , H. Fan , Z. Zhao , H. Wu , M. Xu , R. Shen , Y. Yang , J. Bian , ACS Appl. Electron. Mater. 2020, 2, 3695.

[66] S. Wang , X. Dong , Y. Xiong , J. Sha , Y. Cao , Y. Wu , W. Li , Y. Yin , Y. Wang , Adv. Electron. Mater. 2021, 7, 2100014.

[67] J. C. Li , Y. C. Li , Z. C. Liu , Y. X. Ma , Y. L. Wang , IEEE Electron Device Lett. 2024, 45, 2106.

[68] P. Bousoulas , D. Sakellaropoulos , C. Papakonstantinopoulos , S. Kitsios , C. Arvanitis , E. Bagakis , D. Tsoukalas , Nanotechnology 2020, 31, 45. 10.1088/1361-6528/aba3a1 32634787

[69] H. Luo , L. Lu , J. Zhang , Y. Yun , S. Jiang , Y. Tian , Z. Guo , S. Zhao , W. Wie , W. Li , B. Hu , R. Wang , S. Li , M. Chen , C. Li , J. Phys. Chem. Lett. 2024, 15, 2453. 38407025 10.1021/acs.jpclett.3c03558

[70] F. Faini , V. Larini , A. Scardina , G. Grancini , MRS Bull. 2024, 49, 1059.

[71] C.‐P. Hsiung , H.‐W. Liao , J.‐Y. Gan , T.‐B. Wu , J.‐C. Hwang , F. Chen , M.‐J. Tsai , ACS Nano 2010, 4, 5414. 20707382 10.1021/nn1010667

[72] P. Bousoulas , D. Sakellaropoulos , D. Tsoukalas , Appl. Phys. Lett. 2021, 118, 143502.

[73] C. Tsioustas , P. Bousoulas , G. Kleitsiotis , S. D. Mantas , D. Tsoukalas , Appl. Phys. Lett. 2024, 125, 023508.

[74] M. A. Asoro , J. Damiano , P. J. Ferreira , Microsc. Microanal. 2009, 15, 706.

[75] S. Svanström , T. J. Jacobsson , G. Boschloo , E. M. J. Johansson , H. Rensmo , U. B. Cappel , ACS Appl. Mater. Interfaces 2020, 12, 7212. 31958007 10.1021/acsami.9b20315

[76] X. Zhu , J. Lee , W. D. Lu , Adv. Mater. 2017, 29, 1700527. 10.1002/adma.201700527 28582597

[77] J. C. PérezMartínez , D. MartínMartín , B. Arredondo , B. Romero , Adv. Electron. Mater. 2024, 10, 2400067.

[78] D. Sakellaropoulos , P. Bousoulas , C. Papakonstantinopoulos , S. Kitsios , D. Tsoukalas , IEEE Trans. Electron Devices 2021, 68, 1598.

[79] P. Bousoulas , M. Panagopoulou , N. Boukos , D. Tsoukalas , J. Phys. D: Appl. Phys. 2021, 54, 225303.

[80] X.‐C. Lai , Z. Tang , J. Fang , L. Feng , D.‐J. Yao , L. Zhang , Y.‐P. Jiang , Q.‐X. Liu , X.‐G. Tang , Y.‐C. Zhou , J. Shang , G.‐K. Zhong , J. Gao , Mater. Horiz. 2024, 11, 2886. 38563639 10.1039/d4mh00064a

[81] S. D. Stranks , G. E. Eperon , G. Grancini , C. Menelaou , M. J. P. Alcocer , T. Leijtens , L. M. Herz , A. Petrozza , H. J. Snaith , Science 2013, 342, 341. 24136964 10.1126/science.1243982

[82] X. Yuan , Y. Wang , Z. Xu , T. Zhou , L. Fang , Nat. Commun. 2023, 14, 7110. 37925451 10.1038/s41467-023-42984-y PMC10625607

[83] W. Huang , H. Zhang , J. Tang , Z. Lin , T. Guo , Y. Zhou , S. Jiang , P. Hang , M. Jiao , C. Zhu , L. Wang , D. Yang , X. Yu , X. Li , ACS Photonics 2024, 11, 3095.

[84] J. J. de Boer , B. Ehrler , ACS Energy Lett. 2024, 9, 5787. 39698340 10.1021/acsenergylett.4c02360 PMC11650764

[85] V. Kornijcuk , D. S. Jeong , Adv. Intell. Syst. 2019, 1, 1900030.

[86] X. Liang , J. Tang , Y. Zhong , B. Gao , H. Qian , H. Wu , Nat. Electron. 2024, 7, 193.

[87] C. Tsioustas , P. Bousoulas , G. Kleitsiotis , D. Tsoukalas , Appl. Machine Learning. 2023, 1, 026103.

[88] Y. Yin , S. Wang , R. Weng , N. Xiao , J. Deng , Q. Wang , Z. Wang , P. K. Leung Chan , Small Sci. 2025, 5, 2400415. 40212643 10.1002/smsc.202400415 PMC11935116

[89] L. Sun , Z. Wang , J. Jiang , Y. Kim , B. Joo , S. Zheng , S. Lee , W. J. Yu , B.‐S. Kong , H. Yang , Sci. Adv. 2021, 7, abg1455. 10.1126/sciadv.abg1455 PMC8121431 33990331

[90] G. Kresse , J. Furthmüller , Phys. Rev. B 1996, 54, 11169. 10.1103/physrevb.54.11169 9984901

[91] P. E. Blochl , Phys. Rev. B 1994, 50, 17953. 10.1103/physrevb.50.17953 9976227

[92] J. P. Perdew , K. Burke , M. Ernzerhof , Phys. Rev. Lett. 1996, 77, 3865. 10062328 10.1103/PhysRevLett.77.3865

[93] S. Grimme , J. Antony , S. Ehrlich , S. Krieg , J. Chem. Phys. 2010, 132, 154104. 20423165 10.1063/1.3382344

[94] K. Momma , F. Izumi , J. Appl. Cryst. 2011, 44, 1272.

[95] G. Mills , H. Jonsson , G. K. Schenter , Surf. Sci. 1995, 324, 305.

[96] Z. Jackson , Free spoken digit dataset, https://github.com/Jakobovski/free‐spoken‐digit‐dataset (accessed: November 2025).

