# Supplementary material for: Low‐Power Perovskite‐Based Memristors Enable Fused Reservoir Computing and Neuromorphic Vision with Highly Accurate Color Perception
Source: Small. 2025 Nov 28;22(4):e08167. doi: 10.1002/smll.202508167 (PMC12809200; doi:10.1002/smll.202508167)
Supplement: Supplementary file 1 — Supporting Information [file SMLL-22-e08167-s001.docx]

Supporting Information

Low-power perovskite-based memristors enable fused reservoir computing and neuromorphic vision with highly accurate color perception

Panagiotis Bousoulas,* Spyros Orfanoudakis, Leonidas Tsetseris, Chalalampos Tsioustas, Stefania Skorda, Alexandros El Sachat, Polychronis Tsipas, Athanassios G. Kontos, Thomas Stergiopoulos,* Dimitris Tsoukalas

**Note 1. XRD measurements of the PbI_2_/DEAHCl film**

Figure S1 presents the structural changes in the PbI_2_/DEAHCl film after being annealed at 100 ^o^C. As can be observed, several peaks arise, which clearly indicate the interaction of excess PbI_2_ with the DEAHCl film.





**Figure S1.** XRD diffractogram of the PbI_2_/DEAHCl film annealed at 100 ^o^C.

**Note 2. UPS measurements**

Figure S2 presents the Taus plots for the extraction of the E_g_ values.





**Figure S2.** Tauc Plots of bare CsFAMA with and without DEA passivation.

**Note 3. SEM imaging, contact angle and water test measurements**

Top-view scanning electron microscopy (SEM) images of the bare and modified films were also acquired (Figure S3(a)). These images revealed the existence of more pronounced grains in the post-DEAHCl treated films compared to the bare samples, which appear to consist of layered-like crystals. More importantly, previous pinholes and shunts seem to have been reduced in size, and grain boundaries are less prominent in the treated samples. In conclusion, the SEM images reveal the interaction of DEAHCl with excess PbI_2_ leading to the formation of an overlayer structure on the perovskite surface. Figure S3(b) illustrates the contact angle of distilled water droplets on both reference and modified perovskite films. The modified film exhibits a noticeably larger contact angle compared to the control film. In particular, for the reference film, a contact angle of 71.7° was observed, while for the modified film, a value of 93.2° was extracted. This increase in the contact angle indicates that the modified film has improved water resistance, as it repels water more effectively than the reference film. Another harsh test was conducted by immersing the two films in a vial of water (Figure S3(c)). The result was impressive, as the control film became yellow instantly. On the contrary, the passivated film remained stable for at least 10 minutes. DEAHCl-based passivation creates a thin, waterproof shield on the CsFAMA perovskite layer. This layer effectively protects the perovskite material from deterioration due to moisture exposure in the surrounding environment.

**
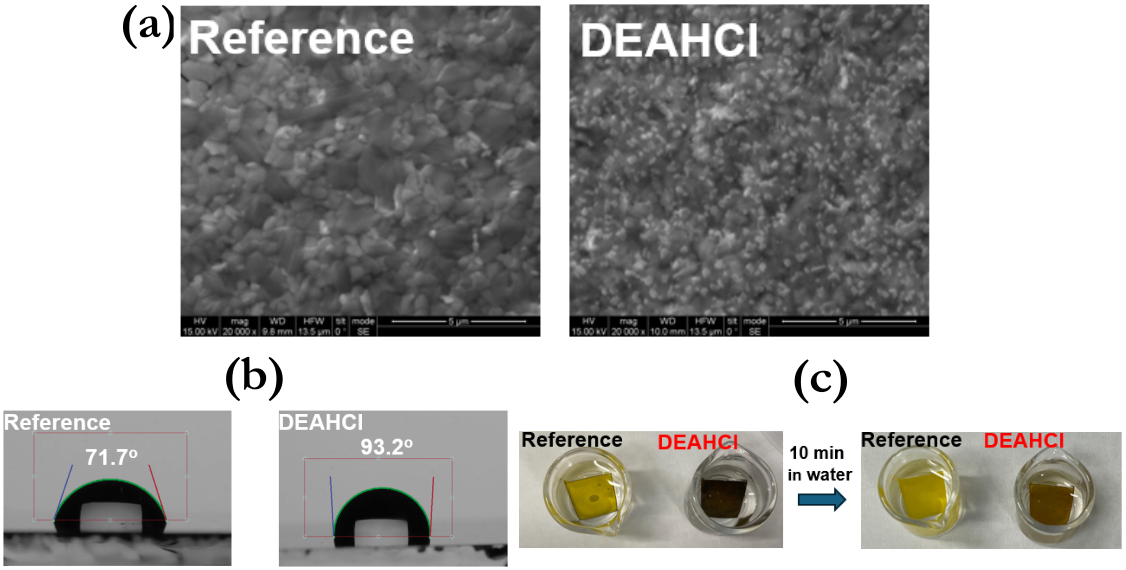
**

**Figure S3.** (a) High magnification top-view SEM images of bare and passivated perovskite films. (b) Contact angle images of bare CsFAMA and DEAHCl-passivated films. (c) Water test results at the moment of immersion and after 10 minutes for both control and passivated films.

**Note 4. XRD measurements after a period of 15 days**

Figure S4 shows the XRD results obtained after a degradation period of 15 days. No structural changes were recorded for the DEAHCl-based film, whereas for the reference film, the PbI_2_ peak was significantly elevated.





**Figure S4.** XRD diffractograms of the degraded ITO/CsFAMA and ITO/CsFAMA/DEAHCl films.

**Note 5. Formation of 2D DEA-FAPbI_3_**


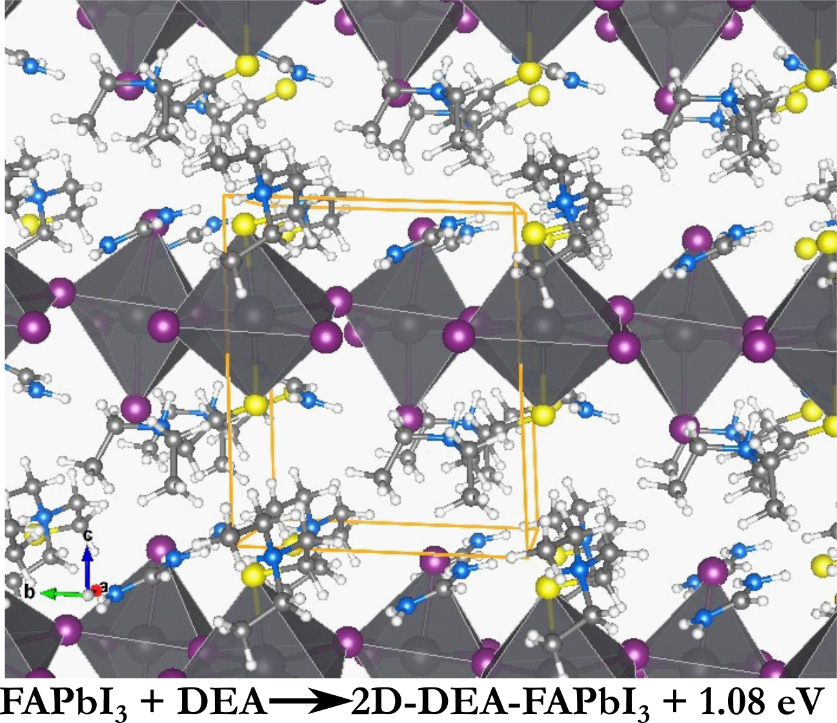


**Figure S5.** A two-dimensional (2D) crystal structure formed through the interactions of DEA molecules with a FAPbI_3_ crystal (Pb: large gray, C: gray, H: white, N: blue, S: yellow, I: purple spheres). Orange lines depict the unit cell. In this structure, the DEA makes a bond through its S end to a Pb atom (the corresponding H atom is transferred to the N site of the DEA). As shown in the above line, the formation of this 2D structure is exothermic with an energy gain of 1.08 eV per chemical formula. The structure has a strong XRD peak at 7.84^o^.

**Note 6. Ag in FAPbI_3-x_Br_x_: individual impurities**


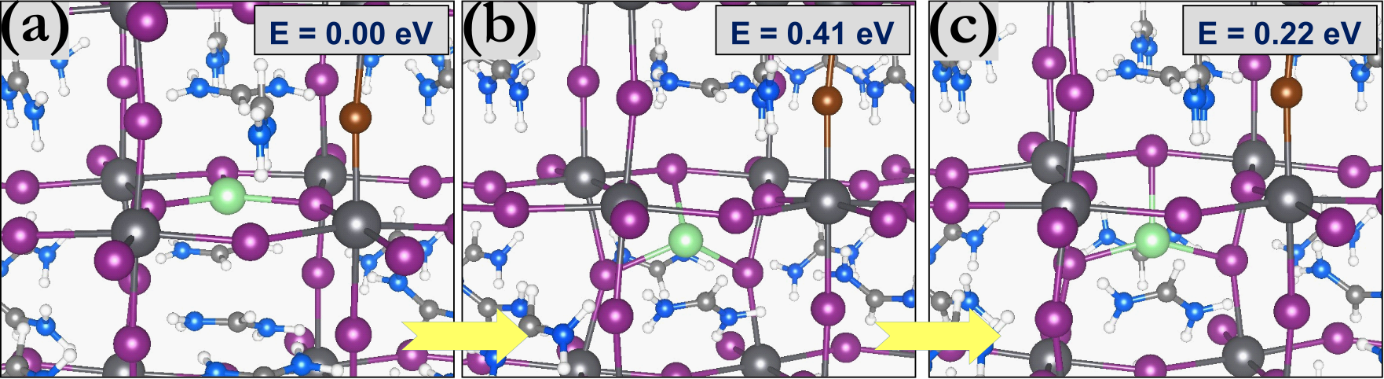


**Figure S6.** An individual Ag impurity in a FAPbI_3-x_Br_x_ crystal (Pb: large gray, I: purple, Br: brown, C: gray, H: white, N: blue, Ag: green spheres). (a) and (c) show locally stable Ag configurations [with (a) being the most stable of all the Ag configurations we examined]. (b) shows the transition state for the process of Ag hopping from (a) to (c). E is the relative energy of each structure. Hence, the energy of (b) gives a value of 0.41 eV for the diffusion barrier for Ag, a relatively low value which indicates that Ag diffusion is activated at room temperature.

**Note 7. Ag in FAPbI_3-x_Br_x_: individual impurities**


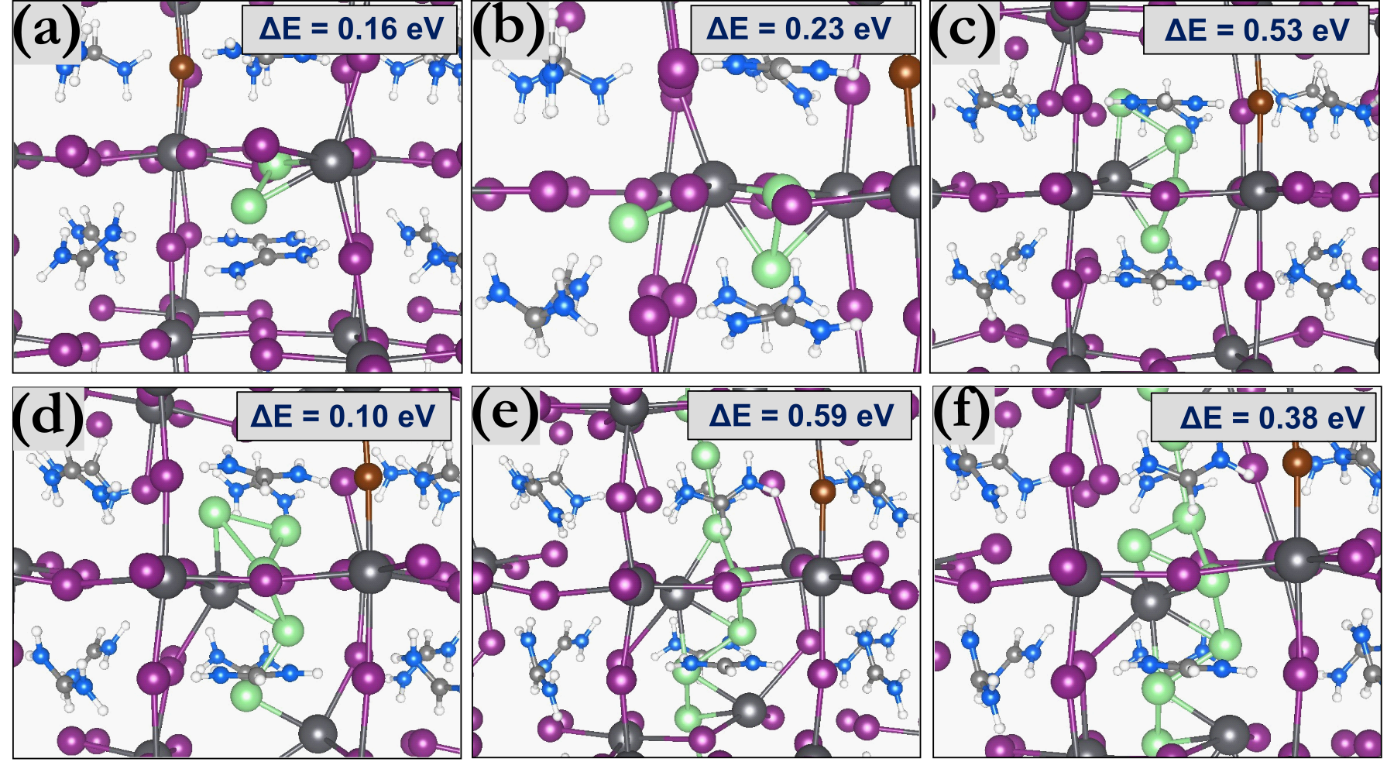


**Figure S7.** Formation of Ag clusters in a FAPbI_3-x_Br_x_ crystal with (a) 2, (b) 3, (c) 4, (d) 5, (e) 6 and (f) 7 Ag atoms. ΔE is in each case the binding energy of the last Ag atom that has been to a cluster with n Ag atoms relative to the energy of a cluster with n-1 Ag atoms and to the energy of an individual Ag impurity. The results show the unequivocal tendency for even larger Ag clusters to be formed. In fact, for the particular supercell employed, the clusters with 6 and 7 Ag atoms form an extended chain within the crystal.

**Note 8. Ag in PbI_2_: individual impurities and clustering**


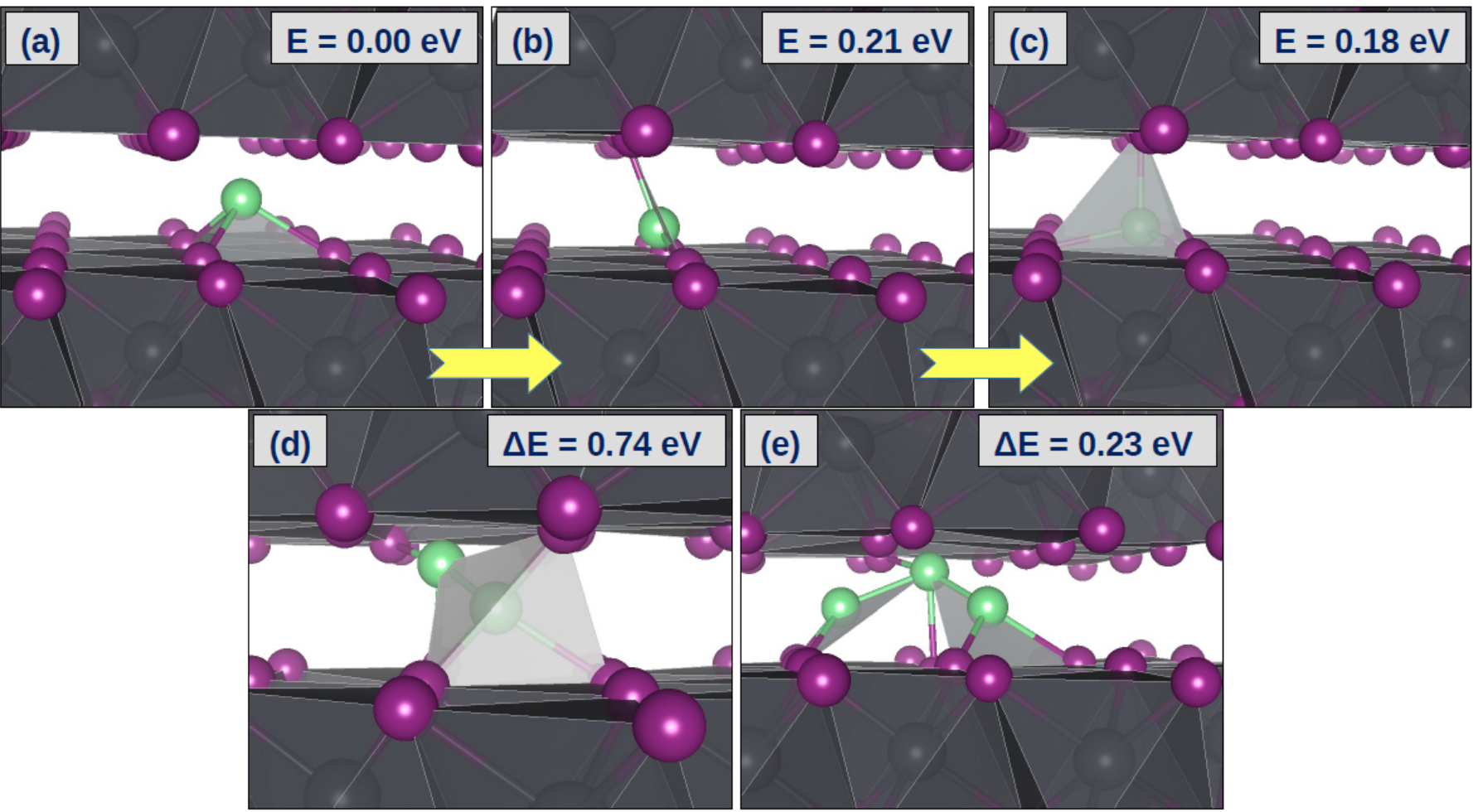


**Figure S8.** An individual Ag impurity in a PbI_2_ crystal (Pb: large gray, I: purple, Ag: green spheres). (a) and (c) show locally stable Ag configurations. (b) shows the transition state for the process of Ag hopping from (a) to (c). E is the relative energy of each structure. Hence, the energy of (b) gives a value of about 0.32 eV for the diffusion barrier for Ag, a relatively low value which indicates that Ag diffusion is activated at room temperature. (d) and (e) show stable clusters with 2 and 3 Ag atoms.

**Note 9. Ag in 2D-DEA-FAPbI_3_**


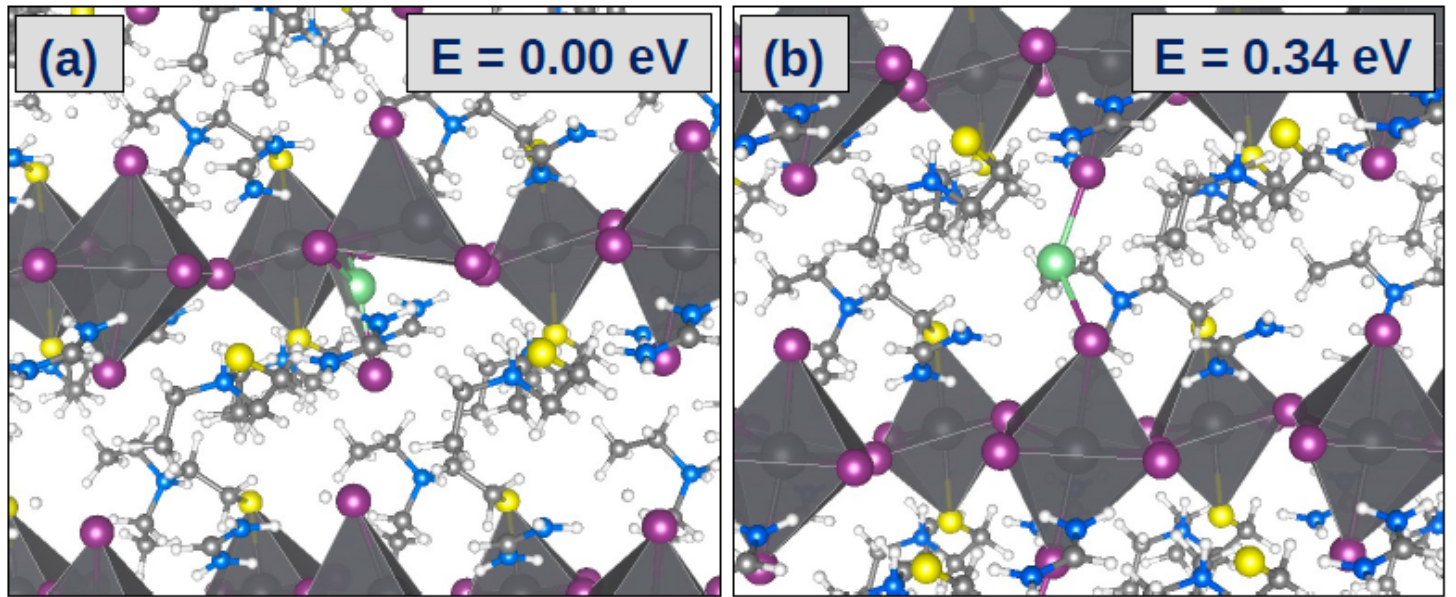


**Figure S9.** An individual Ag impurity in the 2D structure of Fig. S5. The hopping barrier is much larger in this case, about 0.70 eV. So, this 2D overlayer film may block the diffusion of Ag towards the underlying parts of the perovskite substrate.

**Note 10. Ag in low dimensional (DEA)_2_PbI_2_**


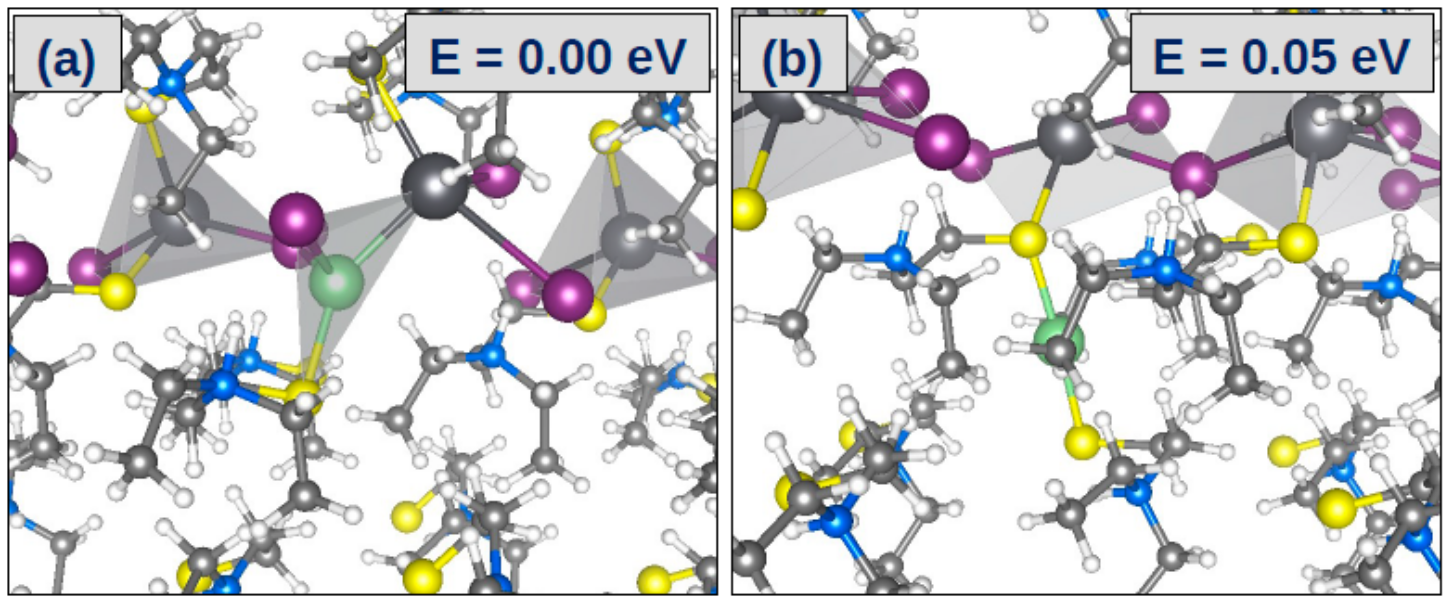


**Figure S10.** An individual Ag impurity in the low dimensional structure of Fig. 1b. The hopping barrier is even larger in this case, more than 1.2 eV. So, this low dimensional overlayer film may block the diffusion of Ag towards the underlying parts of a PbI_2_ crystal.

**Note 11. C-AFM Analysis of Laser-Ablated Interfaces Between Ag and CsFAMA Film**


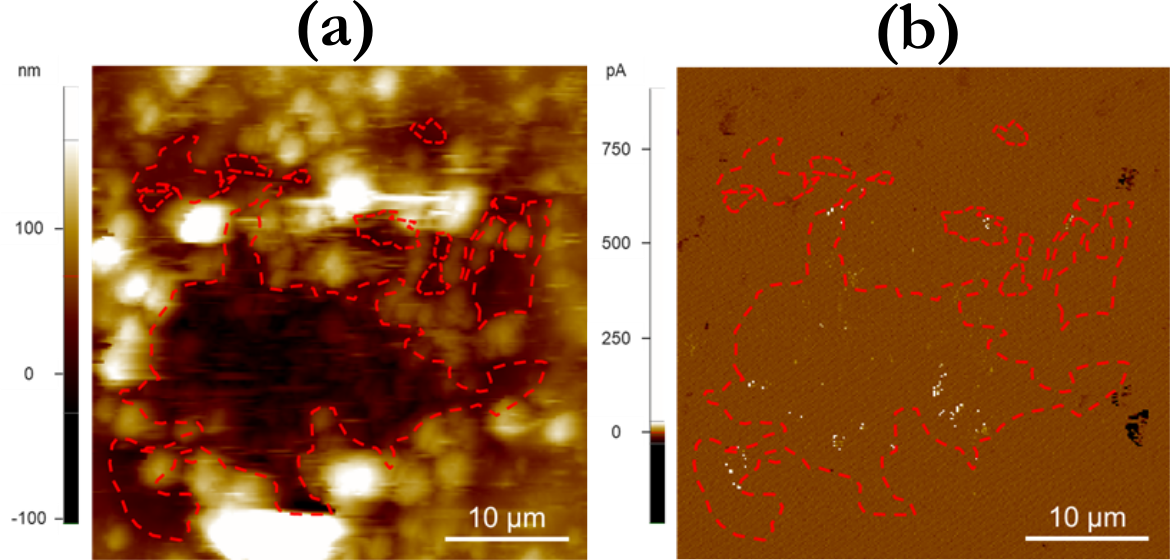


**Figure S11.** (a) Surface topography and (b) current maps of the Ag/DEAHCL/CsFAMA structure following removal of the top Ag electrode by laser ablation. The measurements were performed using C-AFM at a constant bias of 0.5 V, applied from the substrate side, with a scan area of 40 × 40 μm². All data were acquired under high vacuum to prevent contamination from water or hydrocarbons. At 0.5 V bias, CFs appear as bright (white) spots, predominantly within the laser-ablated region of the perovskite film (inside red-dashed areas), while the surrounding areas on the Ag surface remain insulating (brown background).

**Note 12. Device area dependence**

To examine the filamentary concept, devices with different areas of the TEs, in terms of dimensions, were fabricated. The results presented in Figure S12 suggest the total independence of the LRS from the device area, whereas the HRS seems to be affected. Therefore, it can be argued that according to the filamentary theory, the formation of one conducting filament is enough to bridge the two working electrodes and switch the device’s conductance levels. In the case of multiple conducting filaments, a different pattern is anticipated.^[1]^


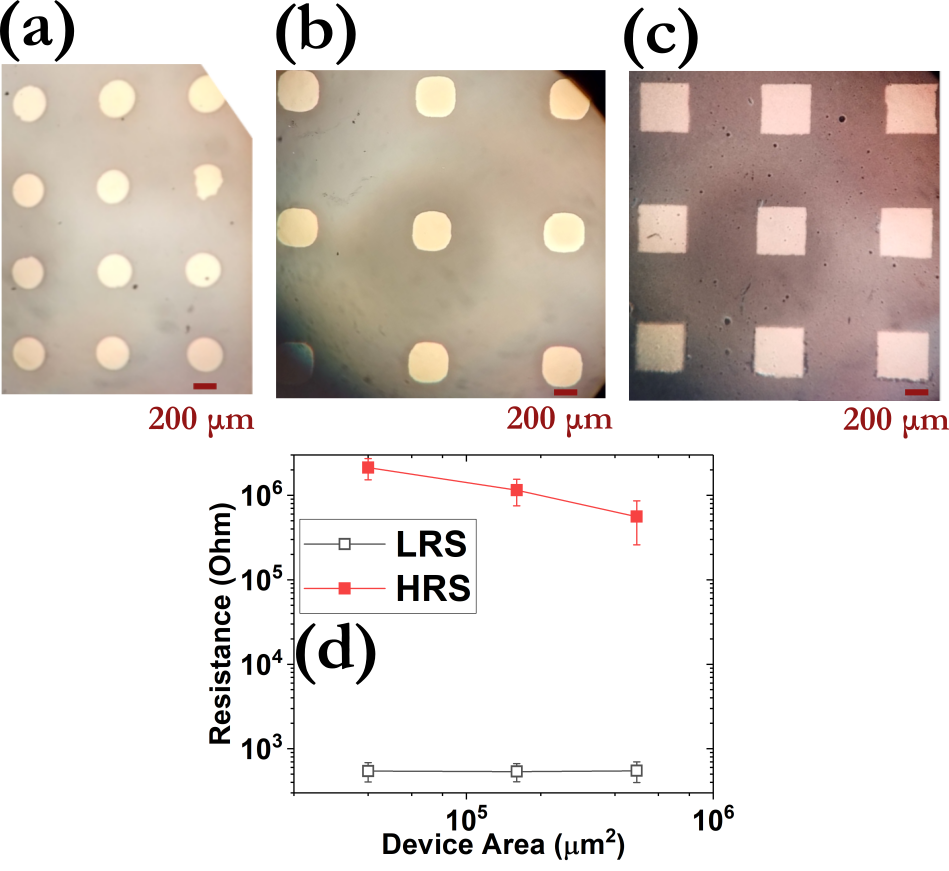


**Figure S12.** Images from an optical microscope illustrating the fabrication of devices with (a) 200 μm, (b) 400 μm, and (c) 700 μm edge dimensions. (d) Distribution of the HRS and LRS for all Samples (read voltage 20 mV).

**Note 13. Geometry of the simulated cell**

Figure S13 presents the CF shape for the materials configurations studied in this work. The pulses were always applied on the TE, while a boundary condition of T = 300 K was enforced for the outermost surface of the electrodes, due to their considerably larger area with respect to the formed CF. The CF was set to be truncated - conical with a diameter of 20 nm at the position z = 640 nm, while a diameter of 6 nm was selected at the position z = 40 nm. This assumption was made to interpret the self-rectification properties of our devices, which could not be explained by solely taking into account the small difference (~0.1 eV) in the respective Schottky barriers between the operating electrodes and the switching material. Although this particular selection seems arbitrary, the simulated outcomes reveal a good consistency with the experimental patterns. Moreover, the radial interference of the outmost surfaces of both CsFAMA and DEAHCl was set total insulating.


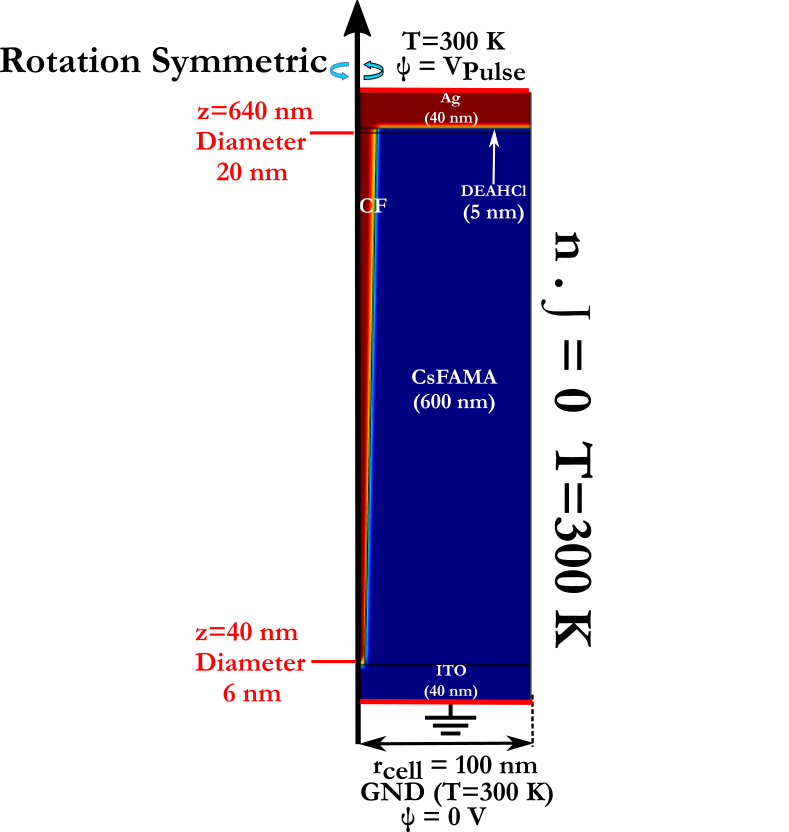


**Figure S13.** Cross-section representation of the simulated memory cell in 2D axisymmetric geometry with the respective boundary conditions for the DEAHCl-based sample. The color denotes the distribution of the CF’s diameter that consists of Ag NCs. The initial values of φ_Β­_ were chosen to reproduce the experimentally recorded operating current values.

**Note 14. Switching model**

The memristive pattern can be simulated by calculating the CF’s effective diameter (φ) by solving the following three differential equations (drift-diffusion, current continuity, and Joule heating):^[2-3]^

 (1)

$\nabla\cdot\sigma\nabla\psi=0$ (2)

$\rho_{m}C_{p}\frac{\partial T}{\partial t}=\nabla k_{th}\cdot\nabla T+\sigma\nabla\left|\psi\right|^{2}$ (3)

where k_B_ is the Boltzmann constant, T denotes the absolute temperature, α is the barrier lowering factor, E_drift_ and E_diff_ represent the energy barriers for ion hopping and diffusion, respectively, ψ refers to the electrical potential, σ is the electrical conductivity, E_S_ is the activation energy of thermophoresis, S is the Soret coefficient, k_th_ is the thermal conductivity, ρ_m_ is the mass density, C_p_ is specific heat, and A, B, and C are constants. The specific values of all parameters can be found in Table S1. The set of equations was solved simultaneously and self-consistently using a numerical solver (COMSOL). Figure S14 depicts the distribution of the electrical conductivity and thermal conductivity functions from the effective diameter of the CF.





**Figure S14.** Dependence of the electrical resistivity and the thermal conductivity as a function of φ for Ag-based CFs.

**Note 15. Simulated profiles for the SET transitions**

The distribution of the applied bias, effective diameter φ_Β_, and total resistance are displayed in Figure S15. A triangular pulse with a rate of 10 mV/s was applied to extract the SET transition. At about 0.25 – 0.3 s the calculated current values start to increase, demonstrating the onset of the former transition. For the applied I_cc_ of 0.1 and 1 μΑ, it can be seen that at t = 2 s, the effective diameter becomes equal with the respective value at t = 0 s, suggesting the manifestation of a threshold switching phenomenon, whereas, for the other two applied I_cc_, no such effect takes place. The simulated profiles smoothly reproduce the respective DC experimental pattern, as well as the recorded transition slopes.

**
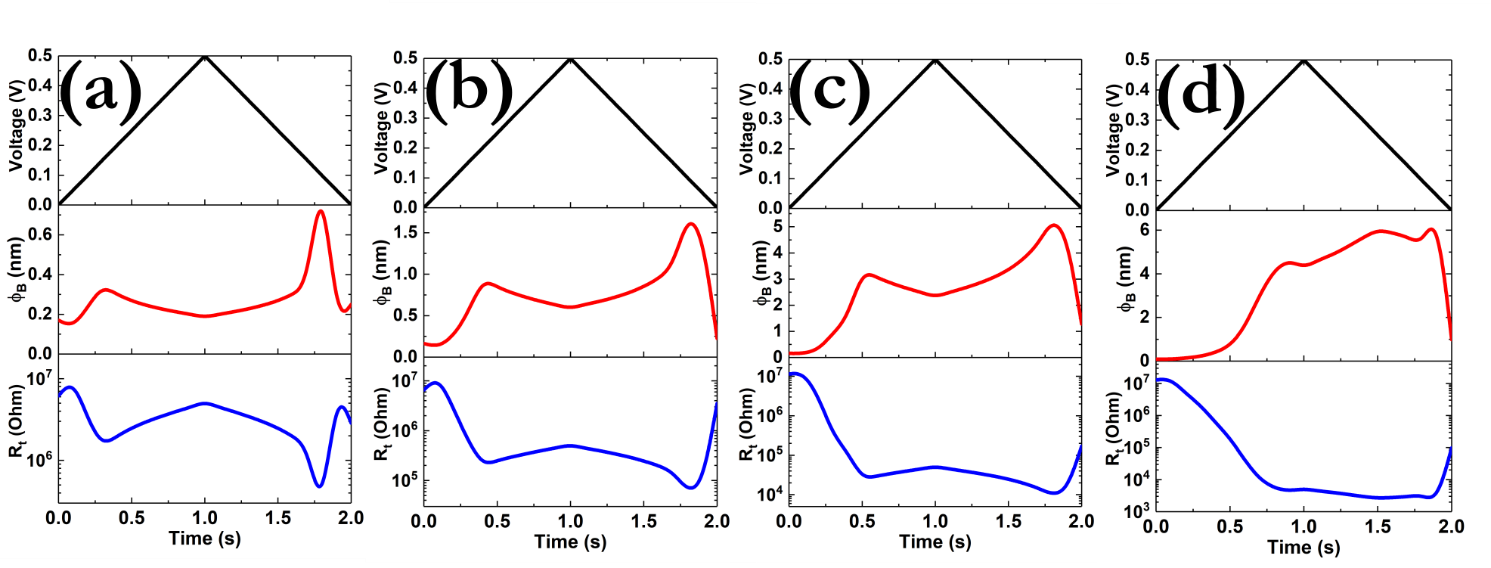
**

**Figure S15.** Distribution of the applied bias, diameter of the CF in the vicinity of the BE, and total resistance during the SET transition for I_cc_ values of (a) 0.1 μA, (b) 1 μA, (c) 10 μA, and (d) 100 μA.

**Note 16. Simulated profiles for the RESET transitions**

Figure S16 shows the distribution of similar characteristics during the RESET process, which were presented above. Smaller CFs were calculated with respect to the application of a positive bias, in terms of the effective diameter distribution, to account for the self-rectification properties. The values of the diameters were selected to provide the respective operating current values of the experimental data. This is attained by choosing a suitable set of fitting parameters, as is reported in Table S1. There is also a physical explanation for the distribution of the effective CF’s diameter near the BE. The development of local high temperatures decisively affects the bottom filament diameter. In other words, a higher local temperature leads to a smaller diameter of the CF during the RESET transition.


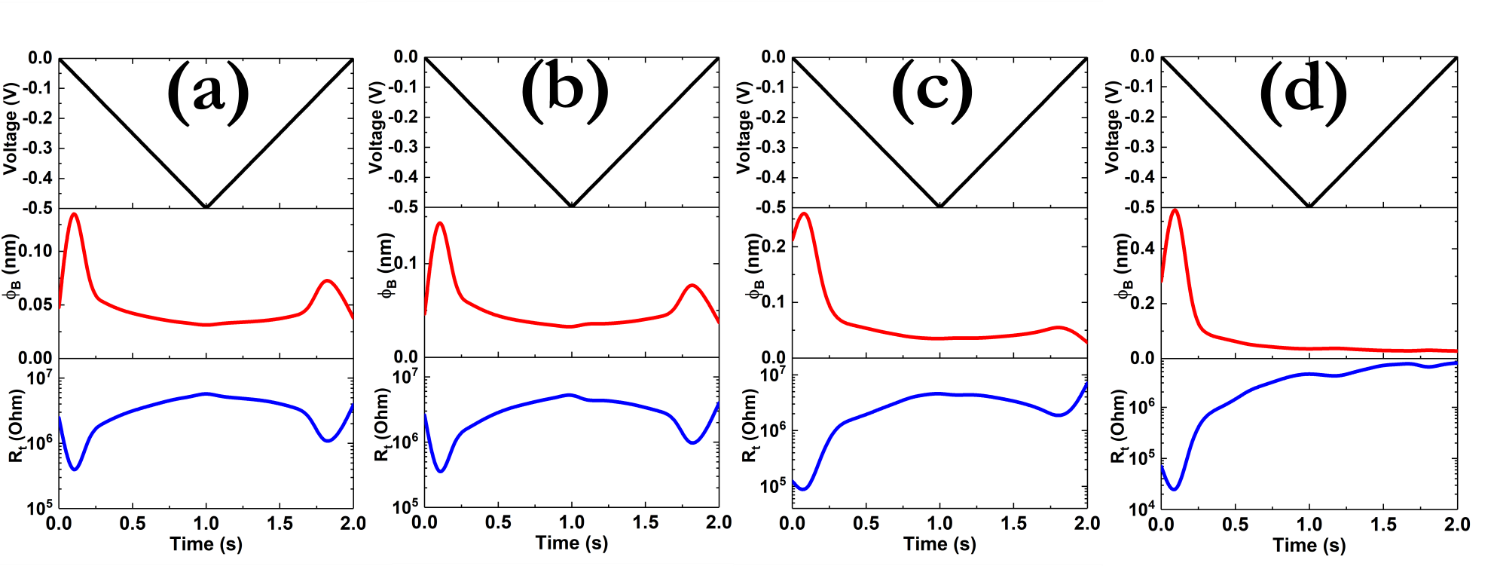


**Figure S16.** Distribution of the applied bias, diameter of the CF in the vicinity of the BE, and total resistance during the RESET transition for I_cc_ values of (a) 0.1 μA, (b) 1 μA, (c) 10 μA, and (d) 100 μA.

**Note 17. Temperature profile**

Figure S17 depicts the formation and rupture processes that take place during device operation. The filament grows toward the anode, eventually creating a continuous conductive path between the two electrodes. As a result, the device switches to the LRS due to the formation of a metallic bridge (Figure S17(a)). The application of a reverse voltage leads to the dissolution of the filament. The filament eventually breaks, disrupting the conductive path (Figure S17(b)). The Joule heating effect plays a key role here considering that the current flowing through the filament generates heat, which eventually weakens it. The generated heat could be as high as 710 K (Figure S17(c)), which could melt the respective constituents of the CF. Taking into account that during the manifestation of the volatile switching mode, the calculated values of the φ_B_ are smaller than 2 nm, the generated heat suffices to locally melt the tip of the CF and induce the creation of a small gap. Hence, the device spontaneously reverts to the HRS.


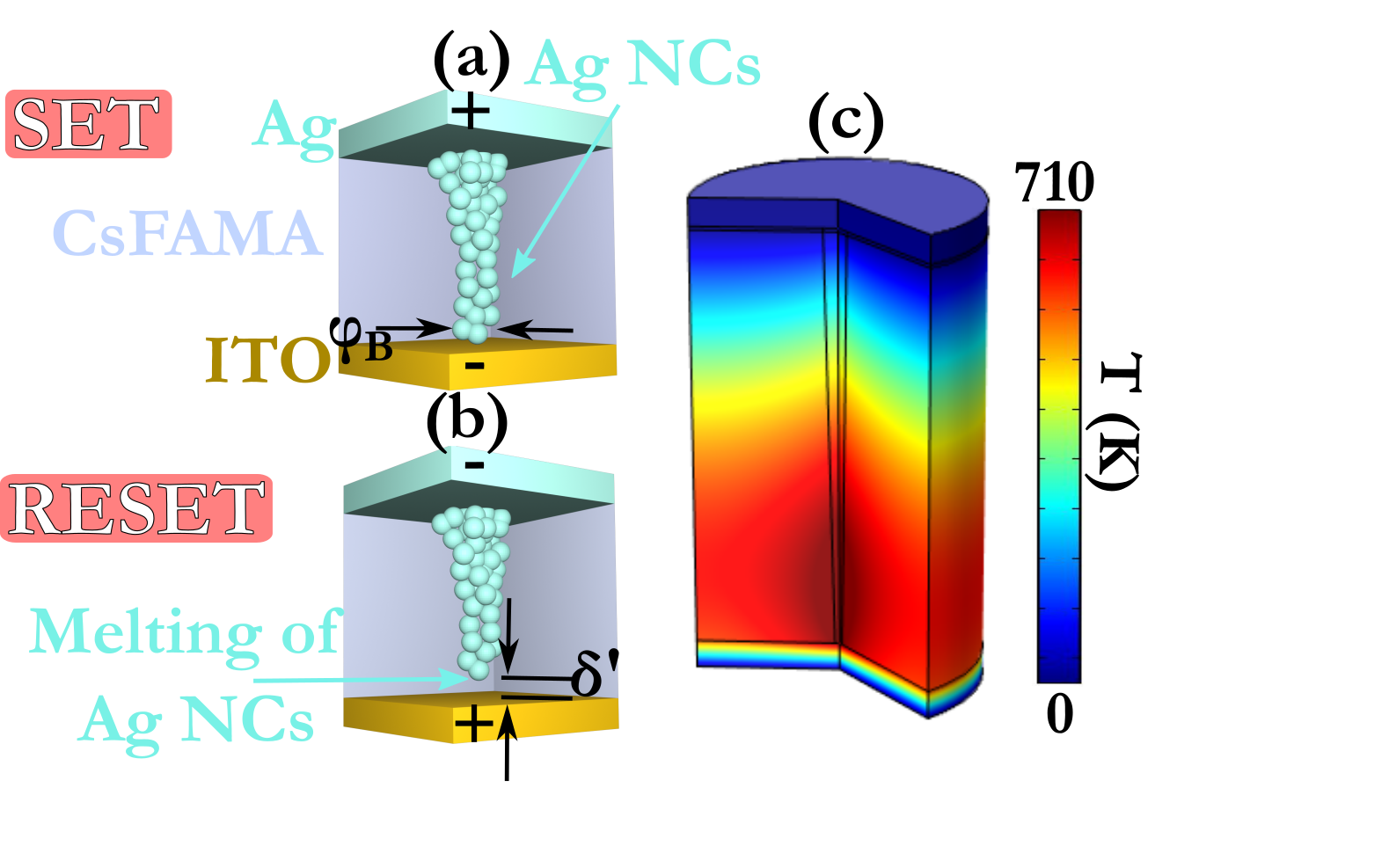


**Figure S17.** Schematic representation of the CF formation and annihilation processes during (a) SET and (b) RESET transitions. (c) 3D calculated maps of the localized temperature distribution at 500 mV bias during switching direction 3.

**Note 18. Relaxation time**

Figure S18 presents the experimentally measured pulsed I-V characteristics during the application of the two-pulsing scheme. Even when the second monitoring pulse is enforced with a delay time of 100 ns, the memory cell is detected at the OFF-state. Similar relaxation times have been reported in our previous work for the SiO_2_-based CBRAMs, pointing out that the respective thermal effect plays a key role in shaping the switching pattern (Ref. [69]). The kinetics of the switching transitions can be also extracted from these measurements. As can be observed, the device switches to the LRS in just 30 ns, while the RESET transition requires a bigger amount of time so as to observe reduced current levels.





**Figure S18.** Pulsed I-V characteristics for the extraction of the relaxation time by enforcing the two pulsing scheme protocol. The first triggering pulse has an amplitude of 0.5 V and 100 ns width, whereas the second monitoring pulse has a lower amplitude of 50 mV and the same width as the previous pulse. The time delay between the two pulses is 100 ns.

**Note 19. Stability of the devices over time**

Figure S19 depicts the measured hysteresis patterns of the reference and DEAHCl-based samples within a period of 2 months. The long-term stability of perovskite-based memristors remains a challenge due to various degradation mechanisms. The degradation of CsFAMA perovskites can primarily result from **environmental factors.** Perovskites are highly sensitive to water. Moisture can penetrate the structure and cause hydration of the perovskite, leading to the formation of hydrated phases. Although oxygen by itself is not highly reactive with perovskites, in the presence of light, oxygen can contribute to photo-oxidation. As a result, reactive oxygen species can be formed, which degrade the organic cations and lead halides. These phenomena can yield increased conductivity. All these effects can be effectively compensated with the incorporation of passivation layers, as can be observed from the measured data. In particular, the reference sample quickly degraded and lost its memory properties, while the DEAHCl-based sample remained fully functional after a period of 2 months.


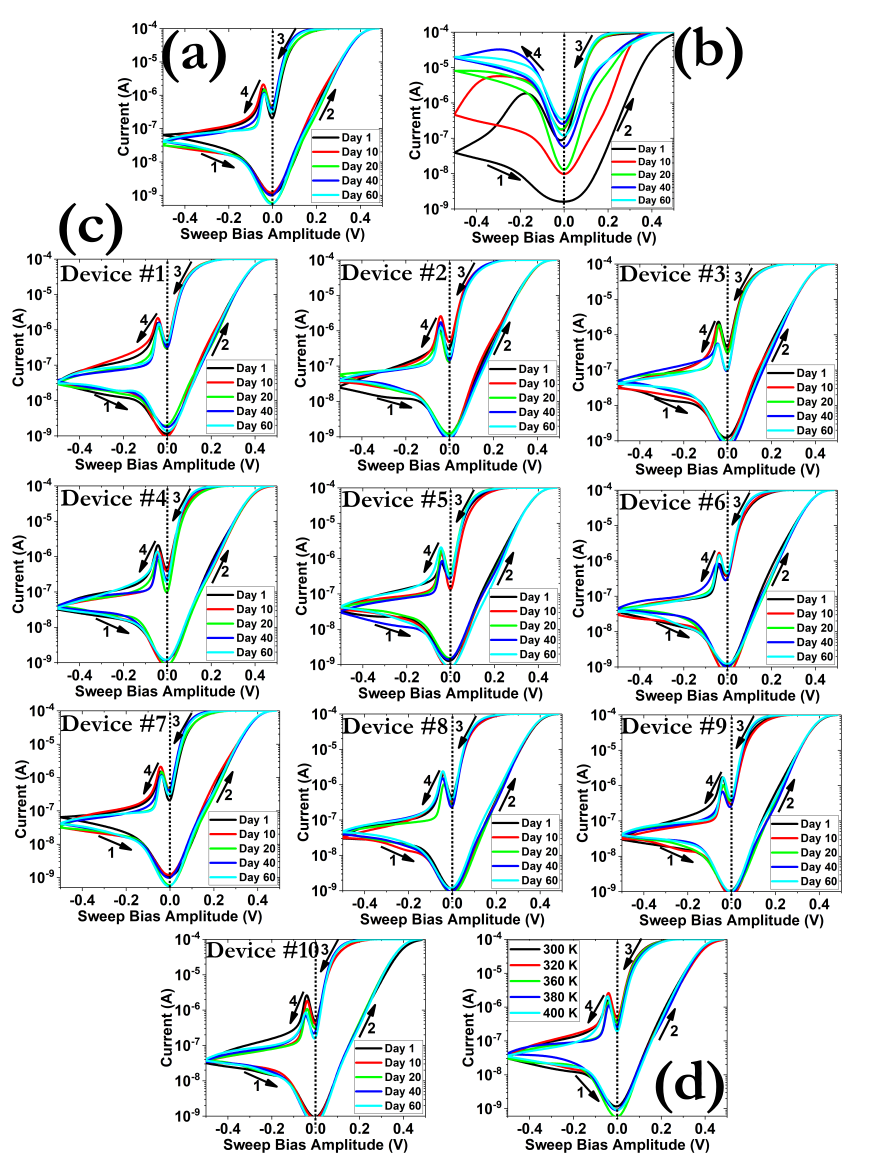


**Figure S19.** I-V hysteresis patterns of (a) DEAHCl-based and (b) Reference samples at different time periods. (c) I-V hysteresis patterns of 10 different devices of the DEAHCl-based sample. In all cases, the sweep rate was 10 mV/s and a constant I_cc_ of 100 μA was enforced. (d) I-V hysteresis patterns at different temperatures.

**Note 20. Energy band diagram configurations**

In perovskite-based resistive switching devices, metal contacts are essential components that significantly affect the device's performance, switching behavior, and reliability. The energy diagram configuration for both samples is displayed in Figure S20, where the whole stack is taken into consideration. From the values of the work function of the respective materials of the ITO and Ag, it is apparent that the self-rectification property of our devices cannot be explained.^[4]^ In addition, for the devices using either TiN or Au as TEs, the respective barrier heights are 0.68 eV and 0.88 eV, which could theoretically explain the small asymmetry in the current values during the application of positive and negative voltages that were experimentally observed in our prototypes.

It is well known that inert or low reactive metals can provide stable interfaces but rely on intrinsic ion migration within the perovskite for switching. The species that could contribute to the manifestation of the resistive switching effect are Pb^2+^ and halogen ions (I^-^ and Br^-^). The migration of FA and MA ions does not lead to the introduction of energy levels within the bandgap of CsFAMA-based perovskites (ref. [32]). For this reason, they were not taken into account here. Although Pb^2+^ ions could potentially lead to the formation of metallic CFs, they require significantly higher energy for their migration with respect to halide ions. The latter could participate in the formation of percolating CFs due to their relatively small diffusivity barriers. However, negligible hysteresis phenomena were observed in our prototypes in striking contrast with the Ag-based sample. It is also interesting to notice that compared to Pb^2+^ ions, Ag^+^ ions are less charged, leading to reduced Coulomb forces during their movement within the perovskite structure. At the same time, Ag^+^ ions possess the smallest atomic radius (126 pm) with respect to the atomic radii of the other mobile ions of FA^+^ (220 pm), MA^+^ (217 pm), Br^-^ (196 pm), and I^-^ (220 pm) (ref. [63]). The Ag^+^ ions can be also formed more easily compared to the halide ions through a simple oxidation process. These effects in conjunction with the small diffusivity barrier of Ag^+^ ions facilitate their migration within the perovskite structure.


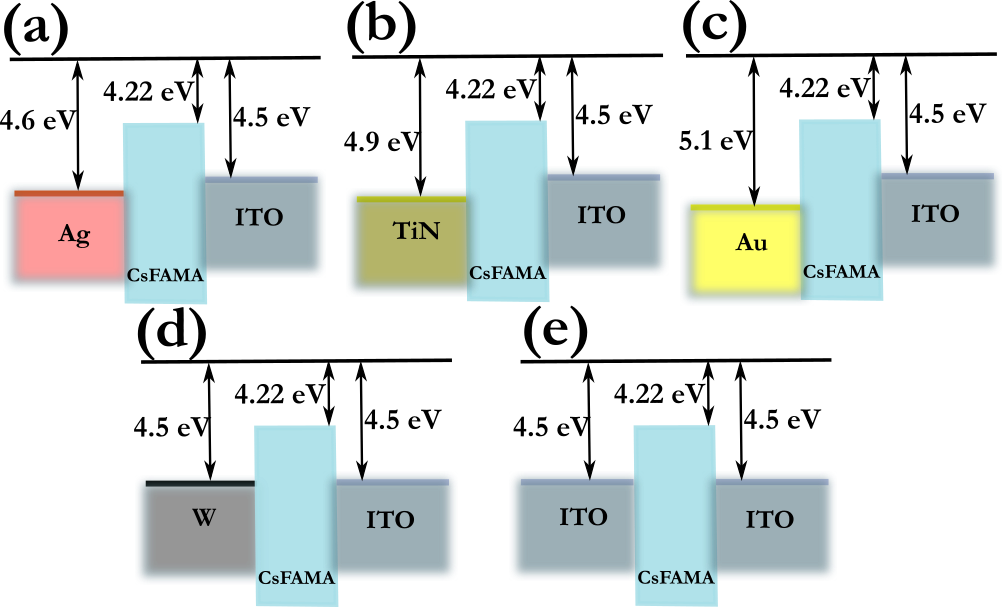


**Figure S20.** Configuration of the energy band diagram using (a) Ag, (b) TiN, (c) Au, (d) W, and (e) ITO as TEs.

**Note 21. Reproducibility of the light-induced reservoir states**

The **reproducibility of reservoir states** refers to the ability of a reservoir computing system to produce consistent internal states in response to identical inputs across multiple runs. This property is crucial for the reliability and robustness of reservoir-based systems. To this end, 20 different devices were tested by applying the same optical pulse sequence, demonstrating reduced variability for all wavelengths (Figure 21).

**
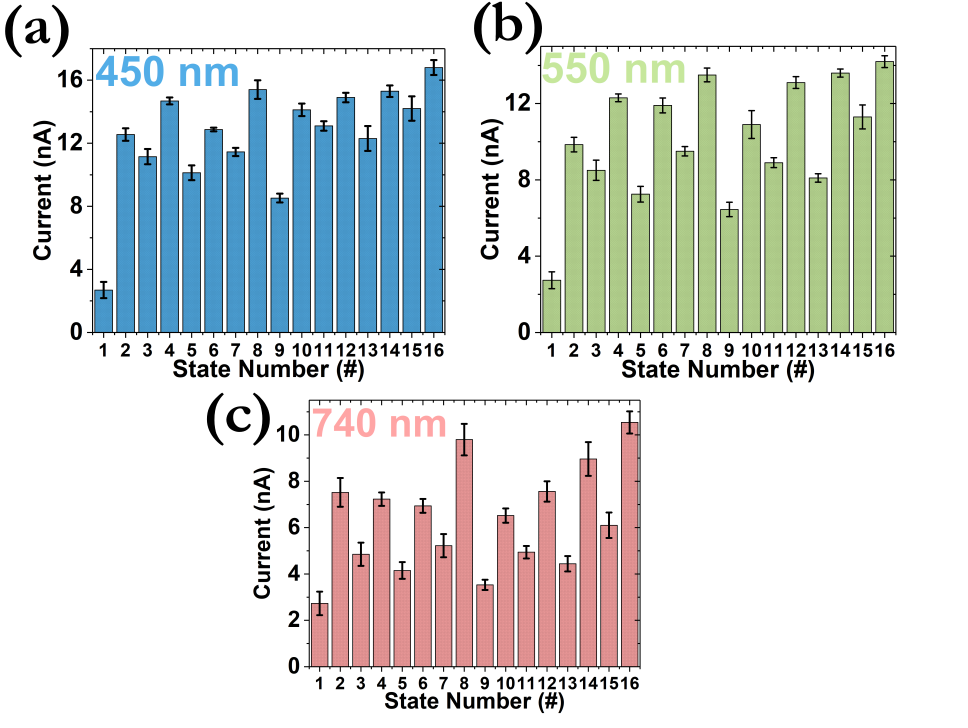
**

**Figure S21.** Current statistics for performing light pulse programming measurements under (a) blue, (b) green, and (c) red light irradiation. The error bar has been extracted by conducting 20 measurements for each state using different devices. All potential combinations of “0000” – “LLLL” were tested. The symbol “0” corresponds to the application of only the read-out pulse, whereas the symbol “L” denotes the application of the light pulse. The state number 1 corresponds to the combination of “0000”, the state number 2 to “000L”, the state number 3 to “00L0”, and so on. The width of the optical and read-out pulses was 1 ms, their interval was 1 s, while the amplitude of the read-out pulse was 20 mV.

**Note 22. Reproducibility of the fused reservoir states**

The reproducibility of the fused reservoir states was also examined using a combination of electrical and optical pulses within the 4-bit coding scheme, yielding excellent results, in terms of device-to-device variation (Figure S22).

**
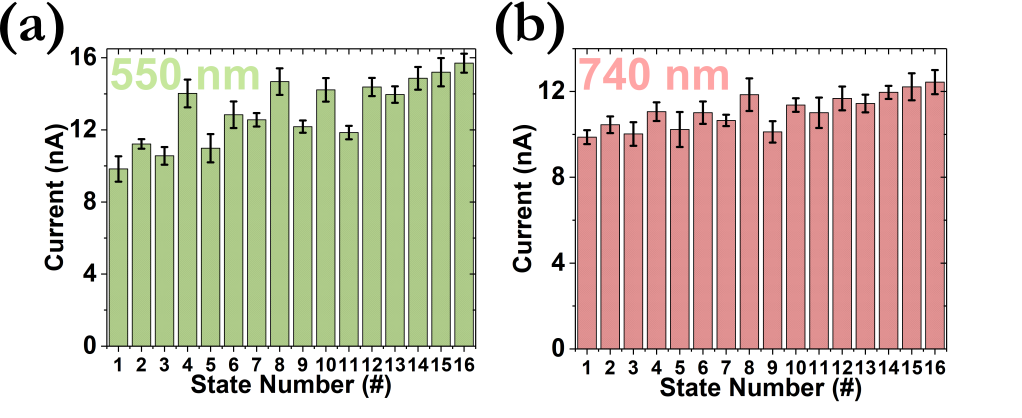
**

**Figure S22.** Current statistics for performing fused programming measurements under (a) green, and (b) red light irradiation. The error bar has been extracted by conducting 20 measurements for each state using different devices. All potential combinations of “EEEE” – “LLLL” were tested. The symbol “E” corresponds to the application of an electrical pulse, whereas the symbol “L” denotes the application of a light pulse. The state number 1 corresponds to the combination of “EEEE”, the state number 2 to “EEEL”, the state number 3 to “EELE”, and so on. The width of the optical, electrical, and read-out pulses was 1 ms, their interval was 1 s, while the amplitude values of the electrical and read-out pulses were 100 mV and 20 mV, respectively.

**Note 23. Image recognition accuracy**

The accuracy of the various fused states considering the three wavelengths that were used in this work, is presented in Figure S23. The combination “EEEE” exhibited the lowest accuracy of 76.82 in recognizing handwritten images from the MNIST database. On the contrary, the fully optical combination of “LLLL” yielded the highest accuracy in all cases. The fused combination of “LLLE” had the second best performance, in terms of accuracy, and was selected for performing our simulations to highlight the capability of our devices to operate under the application of multisensory inputs.

**
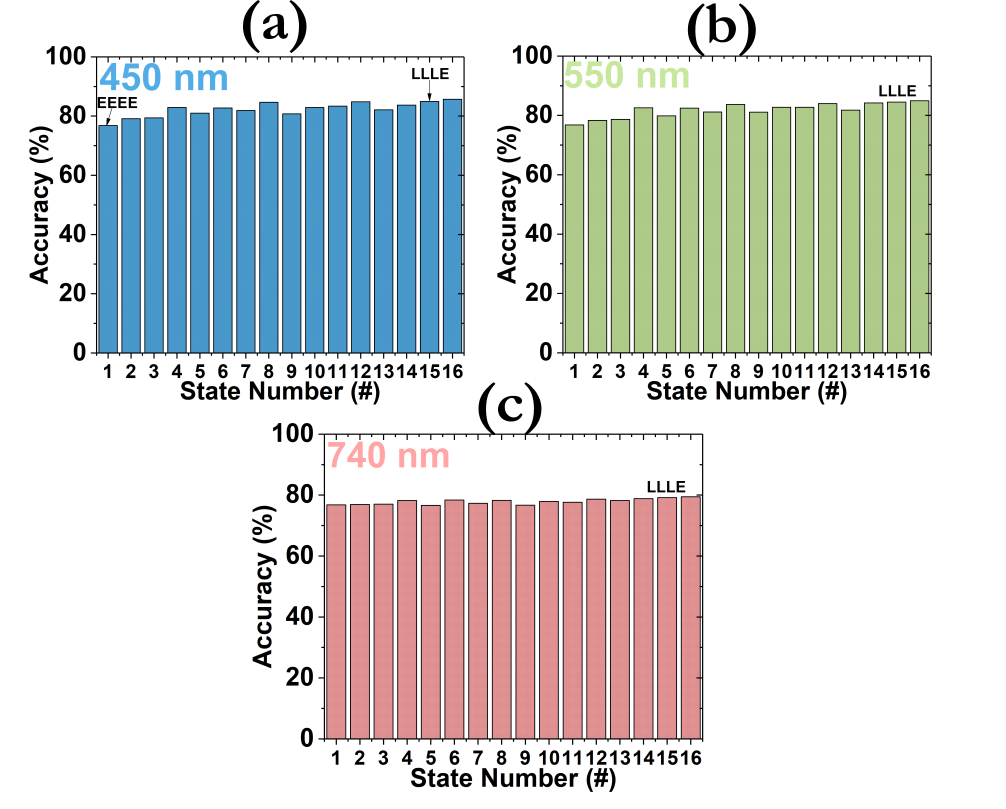
**

**Figure S23.** Recognition accuracies for performing fused programming measurements under (a) blue, (b) green, and (b) red light irradiation. All potential combinations of “EEEE” – “LLLL” were tested. The symbol “E” corresponds to the application of the electrical pulse, whereas the symbol “L” denotes the application of the light pulse. The state number 1 corresponds to the combination of “EEEE”, the state number 2 to “EEEL”, the state number 3 to “EELE”, and so on.

**Note 24. Synaptic weight modulation**

Figure S24 depicts the constant modulation of the output current values under the application of a train of 200 pulses with 1 ms width and 1 s delay time. The results indicate that the devices can respond to both electrical and optical programming with a comparable degree of linearity.

**
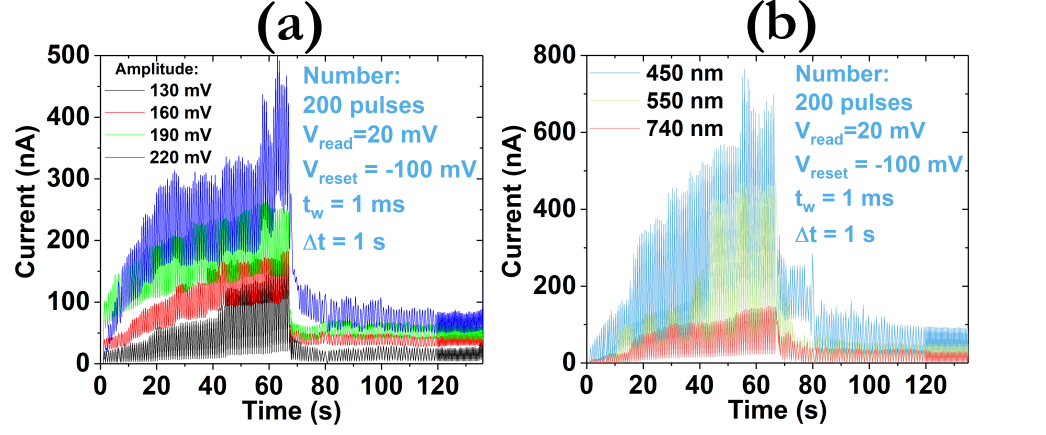
**

**Figure S24.** Continuous modulation of the output current emulating the synaptic potentiation and depression properties of biological synapses under the application of (a) fully electrical pulses and (b) optical pulses for the potentiation process and electrical pulses for the depression. A total number of 100 potentiation and 100 depression pulses were delivered in each case.

**Note 25. Recognition of single-color RGB handwritten digits**

Figure S25 displays the accuracy evolution in recognizing 10 classes of handwritten digits in different background colors, which reaches the value of 88.6%, as well as the respective confusion matrix of the test dataset.


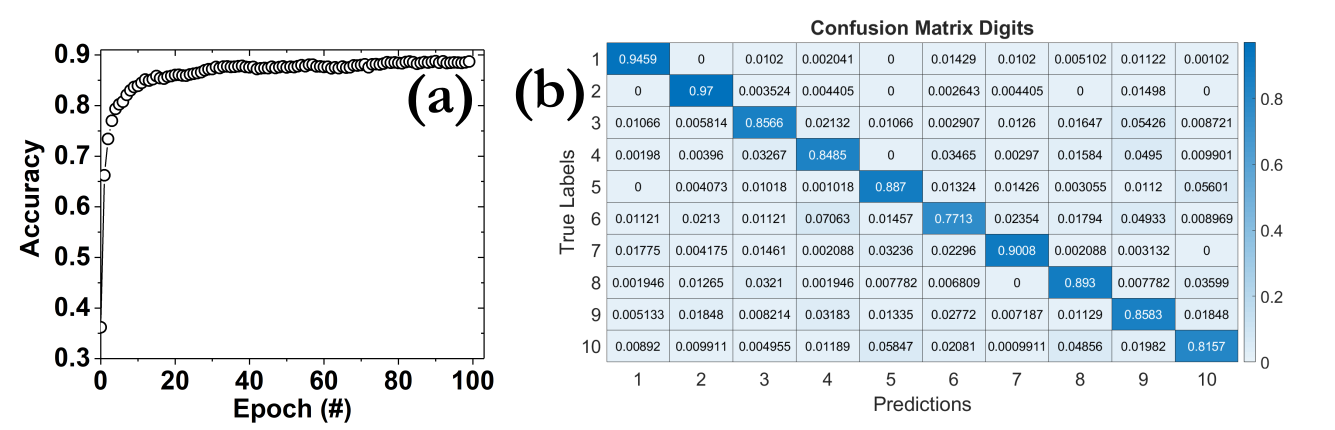


**Figure S25.** (a) Evolution of the training accuracy of the colored MNIST dataset during the classification of 10 handwritten digits in different colors. (b) Confusion matrix of the MNIST classification process used for testing after 100 epochs (accuracy = 87.9%).

**Note 26. Recognition of multi-color RGB handwritten digits**

Figure S26 shows a handwritten digit in a mixture of background colors and the respective confusion matrixes of the test dataset.


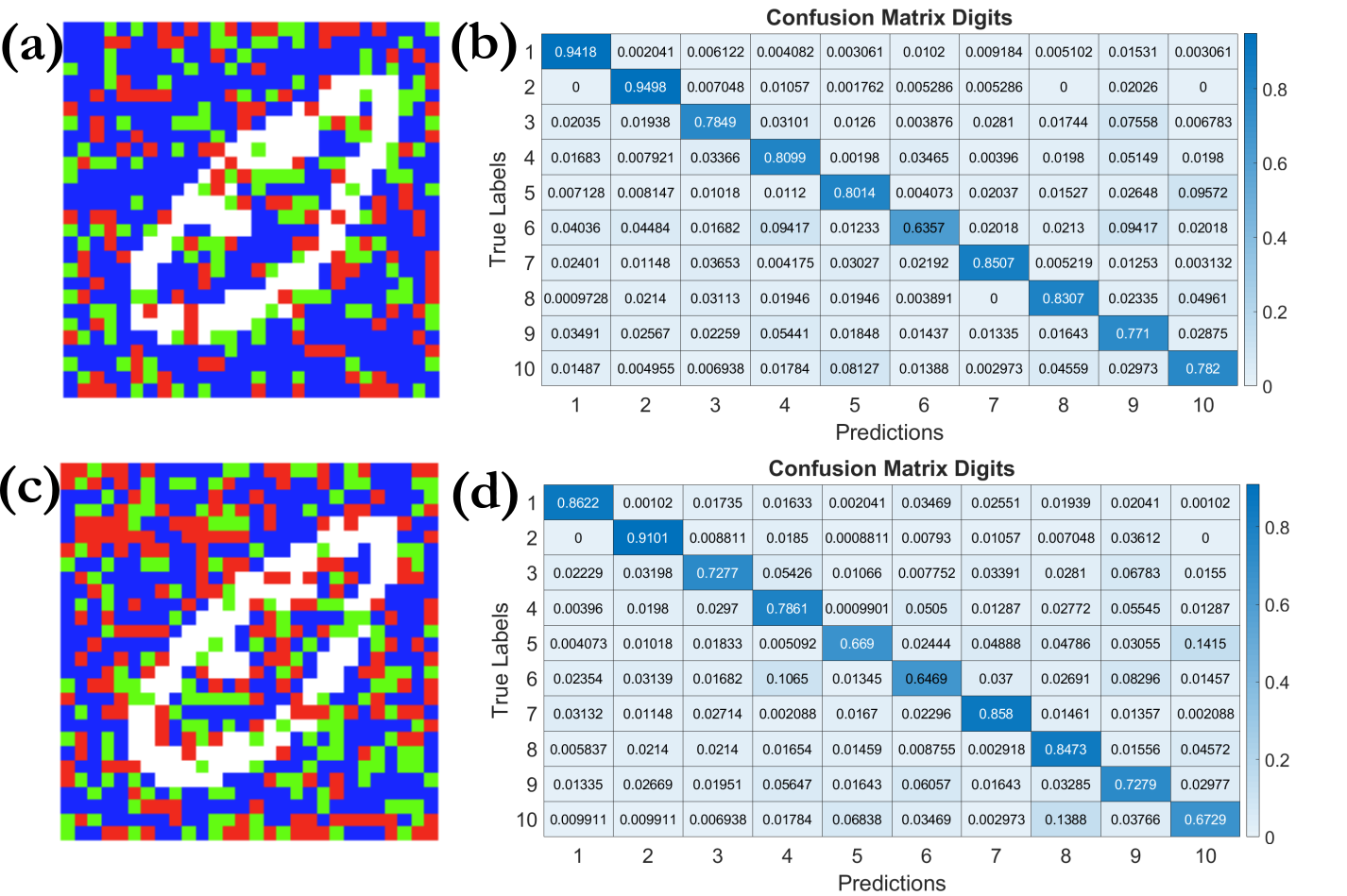


**Figure S26.** Handwritten digit 0 with a noise level of (a) 20% and (c) 30%. Confusion matrixes of the MNIST classification process used for testing after 100 epochs yielding an accuracy of (b) 81.9% and (d) 77.4%.

**Note 27. Recognition of audio-spoken digits**

Figure S27 depicts the respective confusion matrixes of the test dataset that were used for the recognition of the spoken digits. As can be observed, the simulated system was able to fully recognize the speakers with an accuracy of 97.7%.


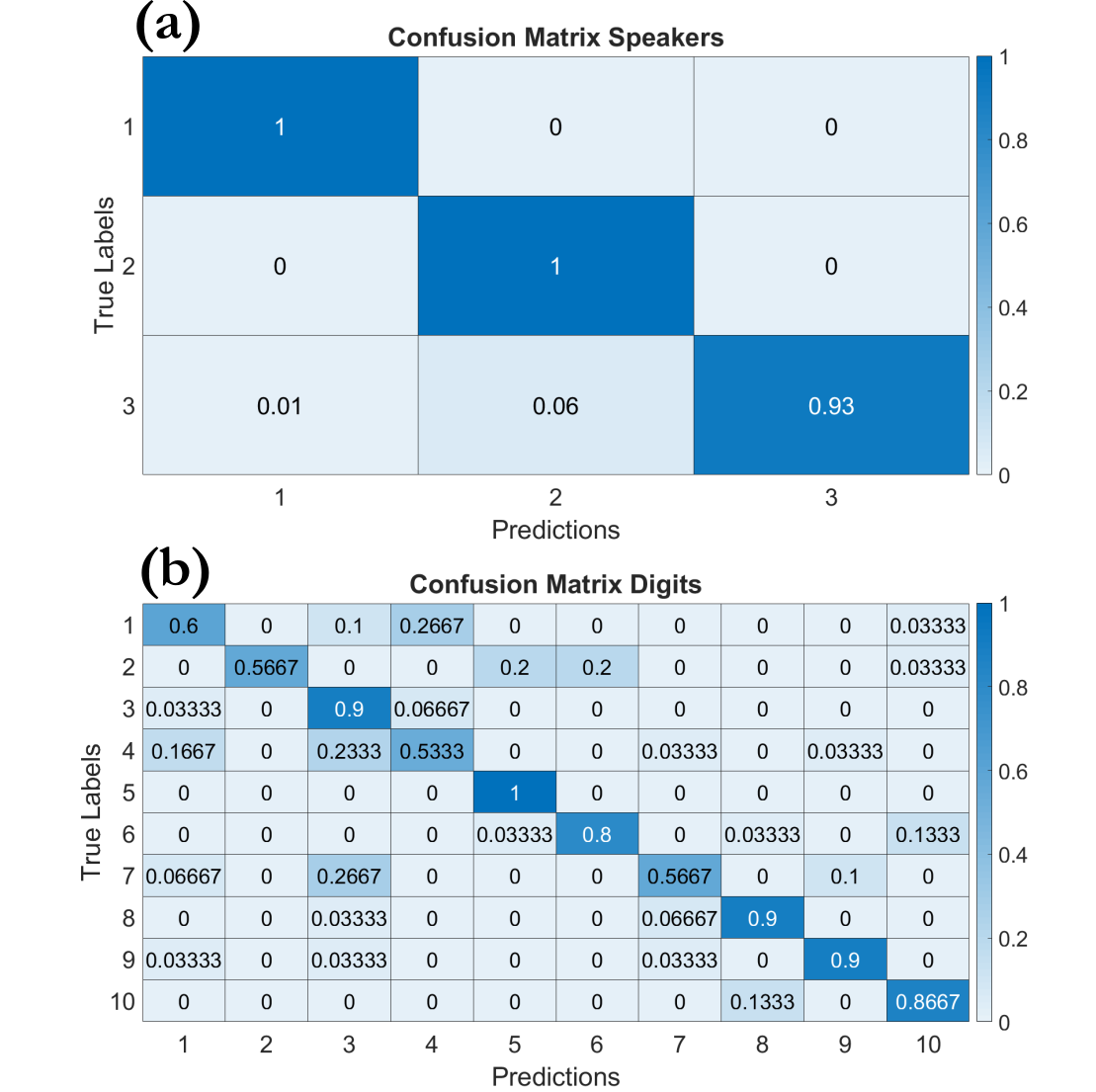


**Figure S27.** Confusion matrixes of the audio classification process used for testing after 100 epochs yielding an accuracy of (a) 97.7% and (b) 76.3%.

Table S1. Model parameters values.

| **Parameter** | **Positive Bias** | **Negative Bias** |
| --- | --- | --- |
| E_drift_ | 0.55 eV | |
| E_diff_ | 0.4 eV | |
| E_s_ | 4 eV | |
| α | 0.25 | 0.95 |
| Α | 9 × 10^-3^ ms^-1^ | 8 × 10^-6^ ms^-1^ |
| Β | 5 × 10^-11^ m^2^s^-1^ | 1 × 10^-12^ m^2^s^-1^ |
| C | 6× 10^-25^ m^3^s^-1^ | 4 × 10^-24^ m^3^s^-1^ |
| ρ_bulk,m_ | Ag: 1.6 × 10^-8^ Ωm  ITO: 5 × 10^-7^ Ωm | |
| ρ_bulk,CsFAMA_ | 1 × 10^3^ Ωm | |
| ρ_bulk,DEAHCl_ | 9 × 10^3^ Ωm | |
| p | 0.5 | |
| λ | 30 nm | |
| γ | 5 × 10^-8^ mV^-1^ | |
| k_th,CsFAMA_ | 5 Wm^-1^K^-1^ | |
| k_th,DEAHCl_ | 4 Wm^-1^K^-1^ | |
| k_th,Ag_ | 398 Wm^-1^K^-1^ | |
| k_th,ITO_ | 4 Wm^-1^K^-1^ | |
| m | Ag: 5 × 10^8^ Wm^-2^K^-1^ | |
| ρ_m_ | Ag: 10.497 kgm^-3^  ITO: 7200 kgm^-3^  CsFAMA: 100 kgm^-3^  DEAHCl: 130 kgm^-3^ | |
| C_p_ | Ag: 238 Jkg^-1^K^-1^  ITO: 341 Jkg^-1^K^-1^  CsFAMA: 110 Jkg^-1^K^-1^  DEAHCl: 120 Jkg^-1^K^-1^ | |

Table S2. Comparison in the performance of various CsFAMA-based devices that have been reported in the literature.

| Material | Switching Voltages (SET/RESET) | Switching Ratio | DC Endurance | AC Endurance | Non-volatile  /Volatile | Ref. |
| --- | --- | --- | --- | --- | --- | --- |
| Cs_x_FA_y_MA_1-x-y_PbI_3-z_Br_z_ | 0.2/-0.2 V | 10^5^ | 10^2^ | 10^10^ | Volatile | [65] |
| Cs_0.06_FA_0.78_MA_0.16_Pb(I_0.92_Br_0.08_)_3_ | 4/-2 V | 10^2^ | 30 | 10^3^ | Non-volatile | [66] |
| Cs_0.05_(FA_x_MA_1−x_)_0.95_PbI_y_Br_3−y_ | 1/-1 V | 10 | 3 | - | Non-volatile | [67] |
| Cs_0.05_(MA_0.17_FA_0.83_)_0.95_Pb(I_0.83_Br_0.17_)_3_ | 3/-3 V | 10^3^ | 10^2^ | 10^4^ | Non-volatile | [68] |
| CsFAMAPbBr_x_I_3-x_ | 1.5/-1.5 V | 10 | - | - | Non-volatile | [S5] |
| Cs_0.05_(FA_x_MA_1−x_)_0.95_PbI_y_Br_3−y_ | 1/-1 V | 10 | 5 | - | Non-volatile | [S6] |
| Cs_0.05_(FA_x_MA_1- x_)_0.95_PbI_y_Br_3-y_ | 1.5/-1.5 V | 10 | 10^2^ | 10^3^ | Non-volatile | [S7] |
| Cs_0.05_FA_0.81_MA_0.14_Pb(I_0.85_Br_0.15_)_3_ | 1/-1 V | 10^2^ | 10^2^ | - | Non-volatile | [S8] |
| CsFAMA | 2/-2 V | - | - | - | Non-volatile | [S9] |
| (Cs_0.05_(FAMA)_0.95_Pb(I_0.85_Br_0.15_)_3_+P(VDF-TrFE) | 1/-1 V | 10^2^ | 10 | - | Non-volatile | [S10] |
| BCP/PCBM/CsFAMA/SnO_2_ | 0.6/-0.6 V | - | - | - | Non-volatile | [S11] |
| Cs_0.05_(FA_x_MA_1_x_)_0.95_PbI_y_Br_3-y_ | 2/-2 V | 10 | 5 | - | Non-volatile | [S12] |
| Cs_0.15_FA_0.85_PbI_3-X_Br_X_ | 1/-2 V | - | - | - | Non-volatile | [S13] |
| **PbI_2-x_DEAHCl/Cs_0.05_FA_0.9_MA_0.05_PbI_2.95_Br_0.05_** | **0.2/-0.05 V** | **10^5^** | **200** | **10^8^** | **Both** | **This work** |

Table S3. Comparison in the performance of various optoelectronic RC-based neuromorphic computing systems that have been reported in the literature.

| Material | λ (nm) | Energy per optical spike | Power of the optoelectronic part of RC | Total power of the RC | Ref. |
| --- | --- | --- | --- | --- | --- |
| α-In_2_Se_3_ | 655 nm | ~10 pJ | - | - | [18] |
| BaSnO_3_ | 375 nm | ~50 pJ | - | - | [19] |
| WS_2_ | 435 nm | ~5 nJ | - | - | [22] |
| h-BN/MoS_2_ | 625 nm | ~10 nJ | - | - | [23] |
| In_2_O_3_·SnO_2_/Nb:SrTiO_3_ | 880 nm | ~1 μJ | - | - | [24] |
| Cs_0.05_(FA_0.83_MA_0.17_)_0.95_Pb(I_0.84_Br_0.16_)_3_ | 780 nm | ~50 pJ | - | - | [84] |
| SnS | 455 nm | ~50 nJ | - | - | [90] |
| UCNPs@SiO_2_/P_3_HT | 980 nm | ~1 nJ | - | - | [S14] |
| MoS_2_ | 405 nm | ~200 nJ | - | - | [S15] |
| ZnO | 625 nm | ~10 pJ | - | - | [S16] |
| InGaZnO/TaO_x_ | 405 nm | ~1.35 nJ | 0.61 μW | 45.78 μW | [S17] |
| ZnO/TiO_x_ | 405 nm | ~500 pJ | - | - | [S18] |
| YO_x_ | 365 nm | ~5 nJ | - | - | [S19] |
| ZnO:N/IGZO | 365 nm | ~10 nJ | - | - | [S20] |
| Organic films | 365 nm | ~0.2 fJ | - | - | [S21] |
| CsPbBr_3_/MoS_2_ | 405 nm | ~4 pJ | - | - | [S22] |
| **PbI_2-x_DEAHCl/Cs_0.05_FA_0.9_MA_0.05_PbI_2.95_Br_0.05_** | **625 nm** | **400 fJ** | **690 nW** | **37 mW** | **This work** |

Table S4. Explanation of the various encoding schemes used for the reservoir computing.

| Input bits | Encoding type | Description |
| --- | --- | --- |
| 0000 | 0000 | Application of only electrical pulses |
| 1111 | 1111 | Application of only electrical pulses |
| EEEE | EEEE (E can be “0” or “1”) – 16 combinations | Mixed – input pulses |
| EEEL | EEEL (L can be “0” or “1”) – 16 combinations | Mixed – input pulses |
| EELE | EELE (E can be “0” or “1”) – 16 combinations | Mixed – input pulses |
| ELEE | ELEE (E can be “0” or “1”) – 16 combinations | Mixed – input pulses |
| LEEE | LEEE (E can be “0” or “1”) – 16 combinations | Mixed – input pulses |
| EELL | EELL (E can be “0” or “1”) – 16 combinations | Mixed – input pulses |
| ELLE | ELLE (E can be “0” or “1”) – 16 combinations | Mixed – input pulses |
| LLEE | LLEE (E can be “0” or “1”) – 16 combinations | Mixed – input pulses |
| ELEL | ELEL (E can be “0” or “1”) – 16 combinations | Mixed – input pulses |
| LELE | LELE (E can be “0” or “1”) – 16 combinations | Mixed – input pulses |
| LEEL | LEEL (E can be “0” or “1”) – 16 combinations | Mixed – input pulses |
| ELLL | ELLL (E can be “0” or “1”) – 16 combinations | Mixed – input pulses |
| LLLE | LLLE (E can be “0” or “1”) – 16 combinations | Mixed – input pulses |
| LLEL | LLEL (E can be “0” or “1”) – 16 combinations | Mixed – input pulses |
| LELL | LELL (E can be “0” or “1”) – 16 combinations | Mixed – input pulses |
| LLLL | LLLL (L can be “0” or “1”) – 16 combinations | Mixed – input pulses |

References

[S1] [S. Asapu](https://ieeexplore.ieee.org/author/37085706760), [T. Maiti](https://ieeexplore.ieee.org/author/37086172527), *IEEE Trans. Electron Devices* **2017**, 64, 8, 3145–3150.

[S2] P. Bousoulas, C. Tsioustas, J. Hadfield, V. Aslanidis, S. Limberopoulos, D. Tsoukalas, *IEEE Trans. Electron Devices* **2022**, 69, 5, 2368–2376.

[S3] A. Bricalli, E. Ambrosi, M. Laudato, M. Maestro, R. Rodriguez, D. Ielmini, *IEEE Trans. Electron Devices* **2018**, 65, 1, 115 – 121.

[S4] L. P. B. Lima, J. A. Diniz, I. Doi, J. G. Fo, *Microelectronic Engineering* **2012**, 92, 86.

[S5] Y. Wang, Y. Xiong, H. Wang, X. Wu, J. Sha, Y. Shang, Y. Zhang, W. Li, S. Wang, *Current Applied Physics* **2023**, 47, 54–59

[S6] H. Guan, J. Sha, Z. Zhang, Y. Xiong, X. Dong, H. Bao, K. Sun, S. Wang, Y. Wang, *Journal of Alloys and Compounds* **2021**, 891, 162096.

[S7] Y. Wang, H. Wang, X. Chen, Y. Shang, H. Wang, Z. An, J. Zheng, S. Wang, *Materials Chemistry and Physics* **2023**, 310, 128488.

[S8] Q. Chen, T. Ha, J. Zeng, Z. He, Y. Liu, J. Sun, M. Tang, Z. Zhang, P Gao, G. Liu, *Nanomaterials* **2022**, 12, 2217.

[S9] H. Sun, H. Wang, S. Dong, S. Dai, X. Li, X. Zhang, L. Deng, K. Liu, F. Liu, H. Tan, K. Xue, C. Peng, J. Wang, Y. Li, A. Yu, H. Zhu, Y. Zhan, *Nanoscale Adv.* **2024**, 6, 559.

[S10] S. Han, T. Ma, H. Li, J. Wu, R. Liu, R. Cao, F. Li, H. Li, C. Chen, *Adv. Funct. Mater.* **2023**, 2309910.

[S11] Q. Chen, Y. Zhang, S. Liu, T. Han, X. Chen, Y. Xu, Z. Meng, G. Zhang, X. Zheng, J. Zhao, G. Cao, G. Liu, *Adv. Intell. Syst.* **2020**, 2000122.

[S12] Y. Wang, Y. Xiong, J. Sha, J. Guo, H. Wang, Z. Qiang, Y. Shang, R. Jia, K. Sun, F. Huang, X. Gan, S. Wang, *J. Mater. Chem. C* **2022**, 10, 1414-1420.

[S13] Y. Li, P. Cheng, L. Zhou, Z. Liu, Z. Zuo, X. Zhan, J. Chen, *Solid-State Electronics* **2021**, 186 108166.

[S14] Y.-B. Leng, Z. Lv, S. Huang, P. Xie, H.-X. Li, S. Zhu, T. Sun, Y. Zhou, Y. Zhai, Q. Li, G. Ding, Y. Zhou, S.-T. Han, *Adv. Mater.* **2024**, 2411225.

[S15] W. Du, C. Li, Y. Huang, J. Zou, L. Luo, C. Teng, H.-C. Kuo, J. Wu, Z. Wang, *IEEE Electron Device Letters* **2022**, 43, 3.

[S16] Y. H. Jang, J.-K. Han, S. Moon, S. K. Shim, J. Han, S. Cheong, S. H. Lee, C. S. Hwang, *Mater. Horiz.* **2024**, 11, 499-509.

[S17] H. Cui, Y. Xiao, Y. Yang, M. Pei, S. Ke, X. Fang, L. Qiao, K. Shi, H. Long, W. Xu, P. Cai, P. Lin, Y. Shi, Q.Wan, C. Wan, *Nat. Commun.* **2025**, 16, 2263.

[S18] H. Huang, X. Liang, Y. Wang, J. Tang, Y. Li, Y. Du, W. Sun, J. Zhang, P. Yao, X. Mou, F. Xu, J. Zhang, Y. Lu, Z. Liu, J. Wang, Z. Jiang, R. Hu, Z. Wang, Q. Zhang, B. Gao, X. Bai, L. Fang, Q. Dai, H. Yin, H. Qian, H. Wu, *Nat. Nanotechnol.* **2025**, 20, 93–103.

[S19] Z. Jiang, Z. Luo, K. Liu, P. Jiao, W. Liu, Y. Liu, *IEEE Trans. Electron Devices* **2024**, 71, 9

[S20] Y. Sun, Q. Li, X. Zhu, C. Liao, Y. Wang, Z. Li, S. Liu, H. Xu, W. Wang, *Adv. Funct. Mater.* **2023**, 2309910.

[S21] X. Wu, S. Shi, B. Liang, Y. Dong, R. Yang, R. Ji, Z. Wang, W. Huang, *Sci. Adv.* **2024**, 10, eadn4524.

[S22] W. Ouyang, Q. Zhang, J. Chen, X. Luo, X. Wang, Y. Chen, F. Yang, Q. Nie, Q. Liu, F. Liu, *Adv. Sci.* **2025**, 2502694.
